# Genome-Wide Transcriptome Profiling, Characterization, and Functional Identification of *NAC* Transcription Factors in Sorghum under Salt Stress

**DOI:** 10.3390/antiox10101605

**Published:** 2021-10-13

**Authors:** Himani Punia, Jayanti Tokas, Anurag Malik, Sonali Sangwan, Anju Rani, Shikha Yashveer, Saleh Alansi, Maha J. Hashim, Mohamed A. El-Sheikh

**Affiliations:** 1 Department of Biochemistry, College of Basic Sciences & Humanities, CCS Haryana Agricultural University, Hisar 125 004, Haryana, India; 005anjusingh@gmail.com; 2 Department of Seed Science & Technology, College of Agriculture, CCS Haryana Agricultural University, Hisar 125 004, Haryana, India; anuragmalikseed@hau.ac.in; 3 Department of Molecular Biology, Biotechnology & Bioinformatics, College of Basic Sciences & Humanities, CCS Haryana Agricultural University, Hisar 125 004, Haryana, India; sonalisangwan03@gmail.com (S.S.); shikhayashveer@gmail.com (S.Y.); 4 Department of Biology, IBB University, Ibb, Yemen; alansi1975@yahoo.com; 5 School of Life Sciences, Medical School (E Floor), Queens Medical Centre, Nottingham NG7 2UH, UK; mahajalal_73@yahoo.com; 6 Botany and Microbiology Department, College of Science, King Saud University, Riyadh 11451, Saudi Arabia; melsheikh@ksu.edu.sa

**Keywords:** differential gene expression, genome-wide association, gene ontology, transcription factors, sorghum, salinity, transcriptomics

## Abstract

Salinity stress has become a significant concern to global food security. Revealing the mechanisms that enable plants to survive under salinity has immense significance. Sorghum has increasingly attracted researchers interested in understanding the survival and adaptation strategies to high salinity. However, systematic analysis of the DEGs (differentially expressed genes) and their relative expression has not been reported in sorghum under salt stress. The de novo transcriptomic analysis of sorghum under different salinity levels from 60 to 120 mM NaCl was generated using Illumina HiSeq. Approximately 323.49 million high-quality reads, with an average contig length of 1145 bp, were assembled de novo. On average, 62% of unigenes were functionally annotated to known proteins. These DEGs were mainly involved in several important metabolic processes, such as carbohydrate and lipid metabolism, cell wall biogenesis, photosynthesis, and hormone signaling. SSG 59-3 alleviated the adverse effects of salinity by suppressing oxidative stress (H_2_O_2_) and stimulating enzymatic and non-enzymatic antioxidant activities (SOD, APX, CAT, APX, POX, GR, GSH, ASC, proline, and GB), as well as protecting cell membrane integrity (MDA and electrolyte leakage). Significant up-regulation of transcripts encoding the *NAC*, *MYB*, and *WRYK* families, *NHX* transporters, the aquaporin protein family, photosynthetic genes, antioxidants, and compatible osmolyte proteins were observed. The tolerant line (SSG 59-3) engaged highly efficient machinery in response to elevated salinity, especially during the transport and influx of K^+^ ions, signal transduction, and osmotic homeostasis. Our data provide insights into the evolution of the *NAC* TFs gene family and further support the hypothesis that these genes are essential for plant responses to salinity. The findings may provide a molecular foundation for further exploring the potential functions of *NAC* TFs in developing salt-resistant sorghum lines.

## 1. Introduction

Soil salinization is a growing concern for agricultural production, severely limiting crop production and geographical distribution [1]. According to the FAO, approximately 23% of cultivated land is subjected to soil salinization, comprising 3.610 ha land and 412 Mha at a global level [2,3]. Urban expansion and population growth cause cultivated land to become more-or-less dry land, necessitating agricultural production on smaller land areas and with limited water resources. By 2050, the global food supply needs to be increased by 70% to feed over 9.8 billion people [4]. Saline soils are predominantly rich in Na^+^ and K^+^ ions. Higher salinity primarily leads to osmotic stress and, subsequently, plant toxicity, with detrimental consequences on all fundamental physiological and molecular functions, such as germination, morphogenesis, photosynthesis, nutrient uptake, and crop yield [5,6,7].

To mount an effective response to cope with salt stress, the sensory modality of plants has evolved several adaptive strategies to perceive and manage oxidative stress [8]. They regulate the uptake of excess ions to avoid ion toxicity, ensuring adequate solutes for an osmotic adjustment and, thus, maintain the turgor pressure and volume of the cells and organelles of growing plants [9]. Yet, whether the plants have receptors or sensors for sodium (Na^+^) ions was not entirely documented. As documented, sodium ions presumably enter the cytoplasm through several cation/anion exchange channels, including vacuolar ion transporters (*H^+^-PPase*), high and low-affinity K^+^ (*HKT*) transporters, channels, and sodium proton antiporters (*NHX*) [10].

Sorghum [*Sorghum bicolor* (L.) Moench] ranks fifth among the world’s most economically valuable cereal crops. It is physiologically classified as a C_4_ plant and is a member of the *Poaceae* family [11]. Because of its wider adaptability to dry and semi-dry regions, moderate drought resistance, and high biomass production, it has gained significant attention. It is an essential food source and a valuable model for understanding the physiological functions and metabolic processes under salt stress in cereals [1]. Because of its greater adaptability to high temperatures, droughts, and salinity conditions in the present global climate change paradigm, the crop is anticipated to become more critical, making it a valuable feed resource [5].

Plants use a multitude of morphological, physiological, and molecular adaptations to defend themselves against abiotic stresses [12,13]. Reactive oxygen species (ROS) are produced at low levels as a byproduct of cellular metabolism. The production of ROS is typically boosted by salinity stress. This can cause metabolic abnormalities, cellular damage, necrosis, and accelerated aging [4,14]. Ionic and osmotic imbalances and oxidative damage are caused by high levels of salinity in roots, resulting in growth retardation, withering, or death [15]. Excess ROS can cause lipid peroxidation, protein oxidation, nucleotide damage, enzyme inhibition, and the activation of programmed cell death (PCD), which are all linked to signaling since the reaction products convey information to downstream processes [1,5,16]. To scavenge high ROS levels, an efficient system of enzymatic and non-enzymatic antioxidants is involved. The enzymatic antioxidants include superoxide dismutase (SOD), peroxidase (POX), catalase (CAT), and ascorbate peroxidase (APX) as well as the enzymes of the ascorbate–glutathione cycle that detoxify ROS. In contrast, non-enzymatic antioxidants include phenolics, ascorbic acid, and glutathione, whose up-regulation suggests a pertinent role of these antioxidants in alleviating salt stress-induced oxidative damage. Establishing a correct K^+^/Na^+^ balance necessitates selective ion absorption, exclusion, and compartmentalization, as well as the synthesis of suitable solutes, including glycine, betaine, and proline [1,5].

Genetic response under salinity occurs in a complex mechanism used by plants to upregulate or down-regulate the production of specific gene products. Transcription factors viz. the *WRKY*, S*NAC*, *MYB,* and *DREB* families are regarded as the virtual switches that directly up-regulate or down-regulate the gene expression. Up-regulation of the *NAC* transcription factor was observed in salinity tolerance in rice and wheat cultivars [16]. In contrast to the ATPase activity, vacuolar pyrophosphatase (*H^+^-PPase*) activity pumps H^+^ via the vacuolar membrane, providing another major driving force for sodium accumulation in the vacuole.

Under high salt concentration, plants respond to Na^+^ in either abscisic acid (ABA)-dependent or abscisic acid-independent pathways. In the ABA-dependent pathways, there is up-regulation of the ABA biosynthesis genes (9-cis-epoxycarotenoid dioxygenase; *NCED3*). Higher accumulation of ABA induces the stomatal closure and expression of down-streaming stress-inducible genes [17]. An excess of Na^+^ ions leads to a quick increase in the cytosolic Ca^2+^ ions, which initiates the Ca^2+^ signaling cascade. It activates the sodium efflux pathway and several other Ca^2+^-binding proteins, such as *SOS1-SOS3*, Calcineurin B-like (CBL) Ca^2+^ binding proteins, and *SOS3*-like calcium-binding proteins (*SCaBP*). *SCaBP5*/*CBL1* knockout mutant plants exhibited salinity and drought hypersensitivity, whereas *CBL1*-overexpressing plants showed drought resistance. Salinity stress also triggers secondary injury responses, where the synthesis and accumulation of compatible osmolytes/solutes allow the cell’s osmotic potential to be lowered, in addition to stabilizing proteins and cellular components. Proline, glycine betaine, sugars, glycerol, sorbitol, and mannitol are the typical osmolytes [18].

High-throughput omics approaches including metabolomics, proteomics, and transcriptomics have recently been established, which are crucial to unveiling the transcriptional regulation and metabolic alterations that are relevant to the governing of salinity tolerance. The present study has attempted a comprehensive account of past developments and current trends related to metabolomics changes at the transcriptomic level and the instigation of molecular interactions, as well as their possible collaboration with one another in sorghum under salt stress. However, comparable studies on genome-wide associations in sorghum under salinity are still, at present, in their infancy. Herein, we aimed to determine the possible regulatory network behind the sorghum tolerance against salinity via metabolomics and transcriptomics studies. The information gained here might help to establish a salt-responsive sorghum network and provide insights and directions for salt-tolerant germplasm maintenance and cereal crop improvement.

## 2. Materials and Methods

### 2.1. Plant Materials, Growth Conditions, and Treatments

**Figure 1:**
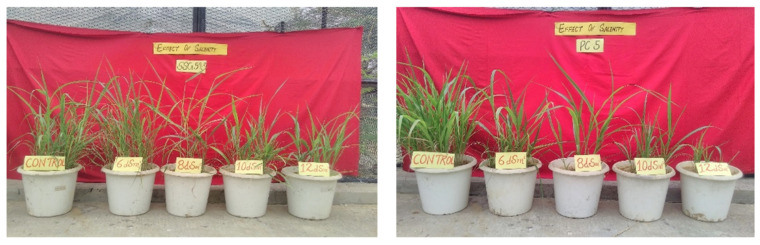
*Sorghum bicolor* genotypes at different salinity treatments at vegetative stage.

**Figure 2:**
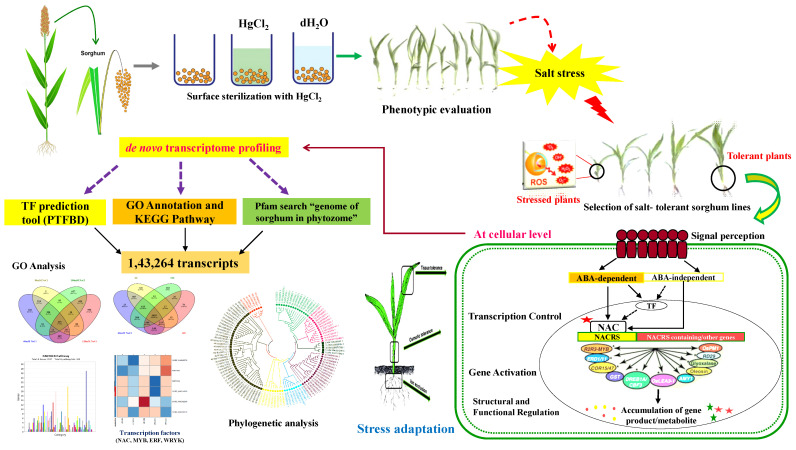
Experimental overview|Two *S. bicolor* genotypes (SSG 59-3 and PC-5) with varied salt tolerance were phenotypically evaluated based on germination studies and then planted in screen house under different salt concentrations. Leaves from 5 plants were pooled and considered a biological replicate, and two such replicates were used in the analysis. Total RNA was isolated from sorghum leaves; sequencing libraries were generated. After library construction, they were sequenced on an Illumina Hiseq-2500 platform to obtain 125 bp/150 bp paired-end reads. The raw data generated were processed and differential expression analysis was performed, followed by phylogenetic analysis, as well as analyses of protein characteristics and gene structure.

The present study was conducted during *kharif* season (the season is popularly considered to start in June and end in October) (2017–2018, 2018–2019, and 2019–2020) in two *S. bicolor* genotypes, viz. SSG 59-3 (Sweet Sudan Grass; source: CCS HAU Hisar, India) as salt-tolerant, and PC-5 (Pant Chari 5; source: GBPUA&T, Pantnagar, India) as salt-susceptible, in the screen house of Department of Biochemistry, CCS Haryana Agricultural University, Hisar, Haryana, India and raised in the plastic pots maintained at 25/20 °C under a 14 h light/10 h dark cycle (Figure 1). All pots were rinsed with an equal volume of water and nutrient solution per the recommended package of practices (POP). The physicochemical properties of the soil used were determined before sowing (Table 1). The pots were saturated with the desired salt levels, i.e., 6 dS m^−1^ (~60 mM NaCl), 8 dS m^−1^ (~80 mM NaCl), 10 dS m^−1^ (~100 mM NaCl), and 12 dS m^−1^ (~120 mM NaCl) (3:1 chloride dominated salinity) in three replicates and control plants. After 48 h of saline treatment, the leaf samples were harvested at the vegetative stage (35 DAS). The samples were quickly frozen in liquid nitrogen and stored at −80 °C for use in RNA extraction (Figure 2). All methods were performed following the protocols set up based on the relevant guidelines and regulations.

### 2.2. Seedling Growth Analysis

Fresh and dry weight and root and shoot length were recorded from control and salt-stressed plants (6, 8, 10, and 12 dS/m). The root and shoot were air-dried and then dried in an oven at 70 °C for 72 h. The dry weights were then recorded in three biological replicates.

### 2.3. Physio-Biochemical Responses and Ion Profiling

Relative water content (RWC) was measured as per Barrs et al. [19]. Leaves were collected, weighed, and immersed in distilled water under diffused light for six hours at a steady temperature. Then, leaf discs were dried in an oven at 80 °C for 72 h for dry weight, which was recorded and calculated as:

$$\mathrm{Relative}\;\mathrm{water}\;\mathrm{content}\;\left(\%\right)=\frac{\mathrm{Fresh}\;\mathrm{weight}\;-\;\mathrm{Dry}\;\mathrm{weight}}{\mathrm{Fully}\;\mathrm{turgid}\;\mathrm{weight}\;-\;\mathrm{Dry}\;\mathrm{weight}}\times 100$$

Chlorophyll estimation was performed by incubating 50 mg of leaf material in 10 mL of DMSO for three h at 60 °C, and other sets of the same amount of leaf were heated at 32 °C for one h in a water bath. After cooling, 10 mL of DMSO was added, and then the absorbance of the solvent was recorded at 663 and 645 nm. The membrane injury index was estimated by measuring the percent of ion leakage into the external aqueous medium to the total ion concentration of the stressed leaves in proportion to the external medium [20]. Two hundred milligrams of leaf and root tissues were heated in de-ionized water at 70 °C, and the solution’s electrical conductivity (EC) was calculated as follows: membrane injury (%) = EC_1_/EC_2_ × 100. Na^+^ and K^+^ content were determined in 50 mg of dried and well-ground plant material. Samples were digested in concentrated H_2_SO_4_/HClO_4_ (9:1), and the clear supernatant was analyzed by mass spectrometry (ICP-MS, Finnigan Element XR, Thermo Scientific, Bremen, Germany).

### 2.4. Biochemical Studies

Samples for biochemical analysis were collected at the vegetative stage (35 days after sowing, DAS) and physiological maturity (95 days after sowing, DAS).

#### 2.4.1. Ascorbate-Glutathione Pool

SOD, CAT, POX, APX, GR, and GPX were determined in a homogenate of 1 g (FW) of leaf tissues, prepared in 5 mL of 100 mM sodium phosphate buffer (pH 7.5) containing 0.25% (*v*/*v*) Triton X-100, 10% (*w*/*v*) polyvinylpyrrolidone and 1 mM phenylmethylsulfonyl fluoride. SOD (EC 1.15.1.1) activity was determined by measuring the inhibition of NBT (nitroblue tetrazolium) reduction at 560 nm [21]. CAT (EC 1.11.1.6) activity was assayed by monitoring the decomposition of H_2_O_2_ at 240 nm [22]. POX (E.C. 1.11.1.7) activity was determined by the oxidation of pyrogallol (ε = 2.47 mM^−1^ cm^−1^) [23]. The APX (EC 1.11.1.11) assay was based on the spectrophotometric monitoring of ascorbic acid oxidation (ε = 2.8 mM cm^−1^) [24]. GPx (EC 1.11.1.9) was assayed by monitoring the continuous regeneration of oxidized glutathione produced by the action of glutathione peroxidase [25]. GR (EC 1.6.4.2)) was assayed by monitoring the non-enzymatic oxidation of NADPH (ε = 6.22 mM^−1^ cm^−1^) [26].

#### 2.4.2. Protein Concentration

The soluble protein was extracted by homogenizing 100 mg fresh tissue as per Lowry et al. [27].

#### 2.4.3. Antioxidant Molecules

Ascorbic acid (ASC) was estimated by homogenizing 500 mg of a sample tissue in 5% (*w*/*v*) metaphosphoric acid [28]. The quantity of ascorbic acid was determined at 530 nm using ascorbic acid’s standard curve (10–100 µg). The total glutathione (GSH + GSSG), reduced (GSH), and oxidized glutathione (GSSG) was estimated by homogenizing 1 g of a sample tissue in 5% (*w*/*v*) sulphosalicylic acid [29]. The reduction rate of 5,5′-dithiobis-(2-nitrobenzoic acid) (DTNB) was monitored at 412 nm.

#### 2.4.4. Compatible Osmolytes

The proline content in the sample tissue was analyzed by homogenizing 500 mg tissue in 5 mL of 3% aqueous sulphosalicylic acid, followed by centrifugation at 5000 rpm for 20 min, and extracted using ninhydrin reagent and toluene extraction [30]. The absorbance was read at 520 nm. Glycine-betaine (GB) estimation was conducted in finely powdered plant material (500 mg) [31]. The dried material was mechanically shaken with deionized water, and the extract was diluted with 2 N sulfuric acids (1:1), centrifuged at 10,000× *g* for 15 min, and absorbance was measured at 365 nm.

#### 2.4.5. Oxidative Stress Markers

Malondialdehyde content was assayed in 250 mg of tissue from control and stressed plants were ground in 2 mL of chilled 1% TCA and centrifuged at 10,000 rpm for 20 min [32]. After centrifugation, the supernatant reacted with 20% (*w*/*v*) trichloroacetic acid (TCA) containing 0.5% thiobarbituric acid to produce pinkish-red chromogen thiobarbituric acid-malondialdehyde (TBA- MDA). Absorbance was measured at 600 nm using the extinction coefficient of 155 mM^−1^ cm^−1^. The relative stress index (RSI) was calculated as the percent proportion of ion leakage into the external aqueous medium to the total ion concentration of the stressed tissue as measured by the external medium EC. RSI (%) = EC_1_/EC_2_ × 100. The hydrogen peroxide (H_2_O_2_) concentration was measured by homogenizing one gram of fresh leaf and root tissue in ice-cold 0.1 M phosphate buffer (pH 7.0), and 40 µL was used in the assay based on the peroxide-mediated oxidation of Fe^2+^, followed by the reaction of Fe^2+^ with xylenol orange at 570 nm [33].

### 2.5. RNA Library Construction and Illumina Sequencing

According to the manufacturer’s protocol, total RNA was isolated from sorghum leaves using the SV Total RNA Isolation System (Promega Corporation, Madison, WI, USA). RNA integrity was checked using Picodrop (Picodrop Ltd., Cambridge, UK) and quantified with an Agilent Technologies 2100 Bioanalyzer (Agilent Technologies, Santa Clara, CA, USA), and sequencing libraries were generated. According to the manufacturer’s protocol, the RNA-seq library was generated from four micrograms of total isolated RNA using a TruSeqRNA sample prep kit (Illumina, San Diego, CA, USA). RiboZero was utilized to remove rRNA transcripts, while poly T oligo coupled magnetic beads were employed to purify mRNA molecules containing poly-A. The Illumina Hiseq-2500 platform was used to obtain paired-end reads of 125 bp/150 bp in two sets of 60 million reads (85% bases have > Q30) for each sample after library construction.

### 2.6. Analysis of Sequencing Data and DEG Estimation

The raw data generated from sorghum samples with biological replicates per treatment were checked initially and processed through FastQC, mapped to the sorghum genome (v.3.1.1) (downloaded from Phytozome v.12.1). Reads were filtered at a quality score (Phred score ≥ 20) for further analysis. Raw readings were pre-processed using Adapter Removal-v2 to eliminate adapter sequences and low-quality bases (v.2.2.0). Ribosomal RNA sequences were deleted from the reads using Bowtie2 (v.2.2.9), BamUtil (v.0.6.7), Sambamba (v.0.6.7), and SAMtools (v.0.1.19) to align the reads with the Silva database (v.1.0.13). All the downstream analyses were based on clean data with high quality (Q-score above 30 (>99.9% correct)) data. The assembled transcripts were annotated by applying NCBI-NR datasets (viride plant) and the UniProt database (Table 2). Cuffdiff (version 2.2.1) in the Cufflinks program was used to estimate genes and transcripts based on the aligned data. *p*-value cutoffs of <0.05 and Log2 fold-change up to (±2) were used individually for up- and downregulated genes in the differential expression analysis. Data were log2-transformed, and TBtools (Beijing, China) were used to generate heat maps from the resulting expression values.

### 2.7. Differential Expression Analysis

Differentially expressed genes (DEGs) were annotated with transcription factors (TFs) based on the annotation file for *Sorghum bicolor* (http://planttfdb.gao-lab.org/msa.php?sp=Sbi&fam=NAC, accessed on 19 June 2021). Using GO enrichment analysis with the PANTHER system, DEGs were submitted to GO term enrichment analysis based on the biological processes, cellular components, and molecular function. For biological pathway analysis of DEGs, MapMan (https://mapman.gabipd.org/mapman-version-3.5.1, accessed on 21 June 2021) was utilized, with the MapMan input file constructed using maize (*Zea mays*) as a model species and a *p*-value cut-off of ≤0.05.

### 2.8. KEGG Pathway Analysis and GO Annotation of DEGs

For functional annotation of the transcripts for the nucleotide-BLAST homology approach, the KEGG pathway and the Uniprot database of *Sorghum bicolor* database were employed (https://blast.ncbi.nlm.nih.gov/Blast.cgi, accessed on 2 July 2021). Transcripts were aligned with a homologous protein from another species if the e-value was below e-5 and the minimal similarity was more than 30%. For antioxidant activities and photosynthetic pathway analysis, the KEGG server was used.

### 2.9. Analysis of Networks between DEGs

The protein–protein interaction influences the function, expression profile, and localization of a specific protein. Here, the STRING (Search Tool for Recurring Instances of Neighbouring Genes) database (http://string-db.org/, accessed on 4 July 2021) version 11.0 was used to investigate the interactions and network analysis of putative candidate genes. A Ramachandran plot was generated for the transcripts using PDBsum to create 3-D structures from the Protein Data Bank (PDB) (https://www.uniprot.org/database/DB-0119, accessed on 19 June 2021).

### 2.10. Phylogenetic Analysis, Protein Characteristics, and Gene Structure

The amino acid sequences of *NAC1* TFs derived from *Zea mays*, *Oryza sativa*, *Oryza nivara*, *Miscanthus lutarioriparius*, *Panicum virgatum*, *Setaria viridis*, *Hordeum vulgare*, and *Digitaria exilis*, along with newly identified *SbNAC1*s, were used to construct a phylogenetic tree. The multiple sequence alignment (MSA) was performed using the E-INS-I option of MAFFT v.7.0 (https://www.ebi.ac.uk/Tools/msa/mafft/, accessed on 18 June 2021). The ambiguous regions within the conserved sequences in the alignment were removed using Gblocks 0.90b. The maximum likelihood (ML) phylogenetic tree of 229 NAC1 protein sequences derived from *Zea mays*, *Oryza sativa*, *Oryza nivara*, *Miscanthus lutarioriparius*, *Panicum virgatum*, *Setaria viridis*, *Hordeum vulgare*, *Digitaria exilis*, and *Sorghum bicolor* was reconstructed using MEGA X (https://www.megasoftware.net/, accessed on 4 July 2021). In total, 1000 replicates were used in the bootstrap analysis. The DNA-binding domain of the *NAC1* TFs was retrieved from PlantTFDB v5.0 (http://planttfdb.gao-lab.org/, accessed on 7 July 2021) using the multiple sequence alignment for the Sorghum bicolor NAC family.

### 2.11. Validation of DEGs Using Quantitative Real-Time PCR (qPCR)

For the validation of DEGs, ten potential candidate genes that were responsive to salt stress were randomly selected from the panel of genes identified in the RNA-seq research using RT-qPCR. qPCR analysis was performed on QuantStudio™ 7 Flex Real-Time PCR (Applied Biosystems, Thermo Fisher Scientific, Waltham, MA, USA) to validate the expression of differentially expressed transcripts. From the total RNA extracted from the leaf samples and treated with DNase I, 2 µg was used as a template for first-strand cDNA synthesis using the iScript cDNA Synthesis Kit (Bio-Rad Laboratories, Inc., Pleasanton, CA, USA) with slight modifications. Primer-BLAST (NCBI) was used for designing the primers, which were custom synthesized from Integrated DNA Technologies, Inc. (Coralville, IA, USA), as listed in Table 2. Prior to sequencing, primer pair specificity was checked using a 2% agarose gel electrophoresis. The pooled and diluted cDNA samples were used for qPCR. Using the NCBI database BLASTN algorithm, the sequencing amplification products were verified and found to be 100% identical, confirming the qPCR primer pairs.

qPCR analysis was performed in a 20 μL reaction volume using Maxima SYBR Green qPCR master mix (2X) (Thermo Scientific) with thermal cycling conditions of 95 °C for 3 min followed by 40 cycles of 95 °C for 30 s, 57 °C for 30 s and 72 °C for 15 s. Expression of the transcripts in control and treated samples were normalized with *Actin-1* and *PP2A* (protein phosphatase 2A subunit A3) as reference genes, and further experiments were performed with *PP2A* as the reference gene. Three biological and five technical replicates from each sample were used for qPCR quantification analysis. An internal reference gene and a target gene were compared, using the comparative C_T_ method (∆∆C_T_ method) [34], to analyze the expression levels of a target gene and the internal reference genes. Melting curve examination of the amplicons verified the qPCR reaction’s specificity. The stability values for each stress-responsive gene were based on the order in which candidate gene stability rankings for the three experimental sets were calculated using three different statistical tools viz. comprehensive stability genes, NormFinder https://moma.dk/normfinder-software/, accessed on 5 January 2020), and geNorm (https://genorm.cmgg.be/, accessed on 5 January 2020).

## 3. Results

### 3.1. Phenotypic and Morphological Variability in Sorghum under Salt Stress

**Figure 3:**
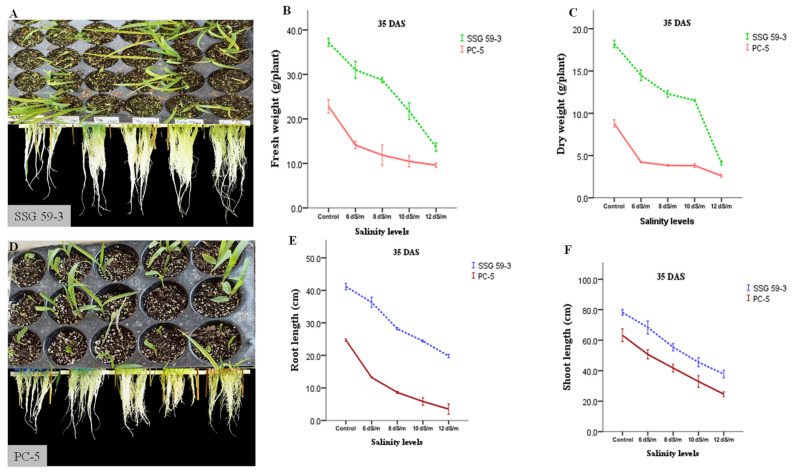
Biomass accumulation and performance of two *S. bicolor* genotypes—SSG 59-3 (salt-tolerant) and PC-5 (salt-sensitive)—under normal (control) and salt-stressed conditions. (**A**) Twenty-one-day-old seedlings and roots of salt-tolerant SSG 59-3 after 48 h of salt exposure. Effect of the salt treatment on (**B**) Fresh weight (FW), (**C**) dry weight (DW), (**D**) 21-day-old seedlings and roots of salt-sensitive PC-5 after 48 h of salt exposure, (**E**) root length, and (**F**) shoot length. All values are the means ± SD of three biological replicates.

Salinity stress significantly reduced the values for several characteristics, with significant differences (*p* < 0.05) observed between the resistant (SSG 59-3) and susceptible (PC-5) sorghum genotypes. Compared to the susceptible genotype, the tolerant genotype SSG 59-3 maintained strong root and shoot growth, with minimal reductions in shoot and root length and fresh and dry weights under salt concentrations (Figure 3).

### 3.2. Trends in Physiological Characteristics

**Figure 4:**
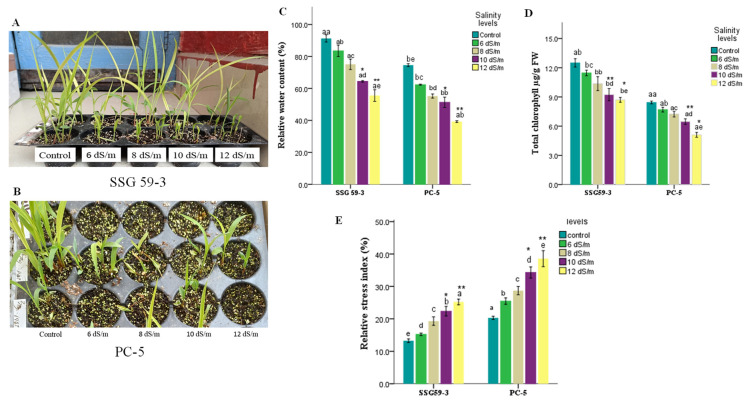
Effect of salt stress on morphological traits of sorghum genotypes. (**A**) Twenty-one-day-old seedlings of salt-tolerant SSG 59-3 after 48 h of salt exposure, (**B**) 21-day-old seedlings of salt-sensitive PC-5 after 48 h of salt exposure. Effect of the salt treatment on (**C**) relative water content (RWC), (**D**) total chlorophyll content, and (**E**) relative stress index. All values are the means ± SD of three biological replicates. Asterisks represent significant (*) and highly significant (**) differences among different treatments. ^a–e^ Values with different superscripts in the same row are significantly different at *p* < 0.05.

Salinity significantly (*p* < 0.05) reduced the leaf relative water content (RWC) in both genotypes, but the decline was more prominent in the susceptible as compared to the tolerant genotype (Figure 4). A similar trend of results was observed for total chlorophyll content, with a more significant decrease in PC-5 (45.5% reduction) than SSG 59-3 (17.2% reduction) and the control condition (Figure 4). The relative stress index varied among the studied genotypes under salinity (*p* < 0.05), with a 3.9-fold increase in the stress index ratio of PC-5, but it was less than half than that obtained for SSG 59-3 (1.02-fold) (Figure 4), indicating that SSG 59-3 possesses a better membranous network and resistance to uncontrolled plants in terms of ion leakage under salt stress.

### 3.3. Ion Dynamics under Salt Stress

**Figure 5:**
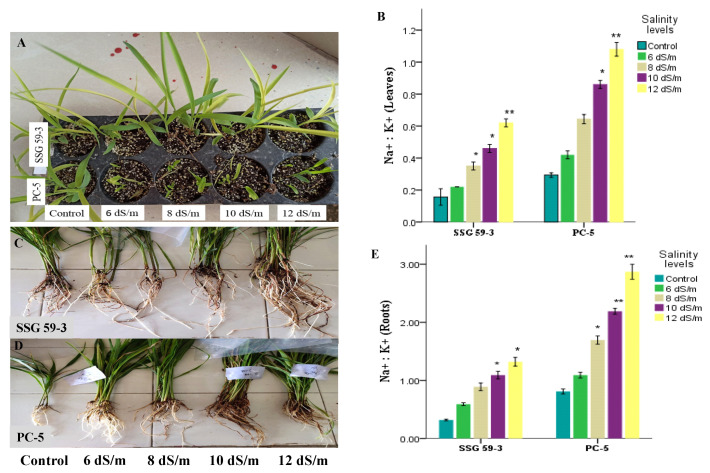
Effect of salt stress on ion profiling of sorghum genotypes. (**A**) Seedlings of SSG 59-3 (salt-tolerant) and PC-5 (salt-sensitive) genotypes after 48 h of salt exposure, (**B**) Na^+^/K^+^ ratios in leaves, (**C**) root biomass of SSG 59-3 (salt-tolerant), (**D**) root biomass of PC-5 (salt-sensitive) genotype, and (**E**) Na^+^/K^+^ ratios in roots. All values are the means ± SD of three biological replicates. Asterisks represent significant (*) and highly significant (**) differences among different treatments.

The Na^+^ concentrations had a significant (*p* < 0.05) effect on root and shoot biomass in both tolerant and susceptible genotypes than control plants, while, in the treated plants, the sensitive genotype, PC-5 (4.92), accumulated a significantly higher Na^+^ ion concentration in the shoot than the tolerant genotype, SSG 59-3 (1.62) (Figure 5). Moreover, the tolerant genotype SSG 59-3 maintained higher K^+^ concentrations in the shoot, while this was considerably reduced in the susceptible genotype PC-5. The K^+^ ion concentrations decreased at 120 mM NaCl in both cultivars, and this was most prominent in the susceptible ones in roots. Based on Na^+^ and K^+^ ions concentrations, the Na^+^/K^+^ ratios in the root and shoot were significantly affected in both genotypes, and were higher in the susceptible PC-5 genotype than the tolerant SSG 59-3.

### 3.4. Antioxidative Enzymes

**Figure 6:**
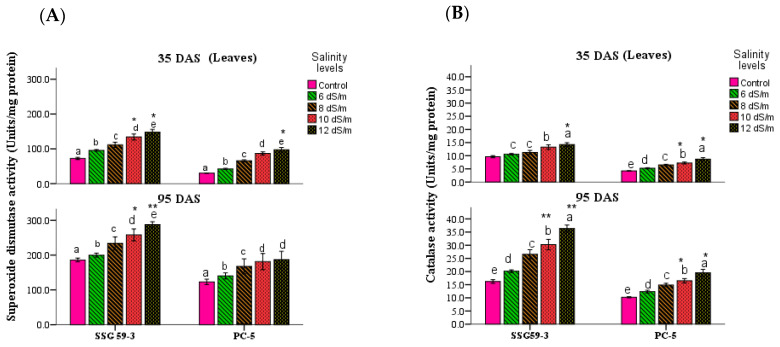

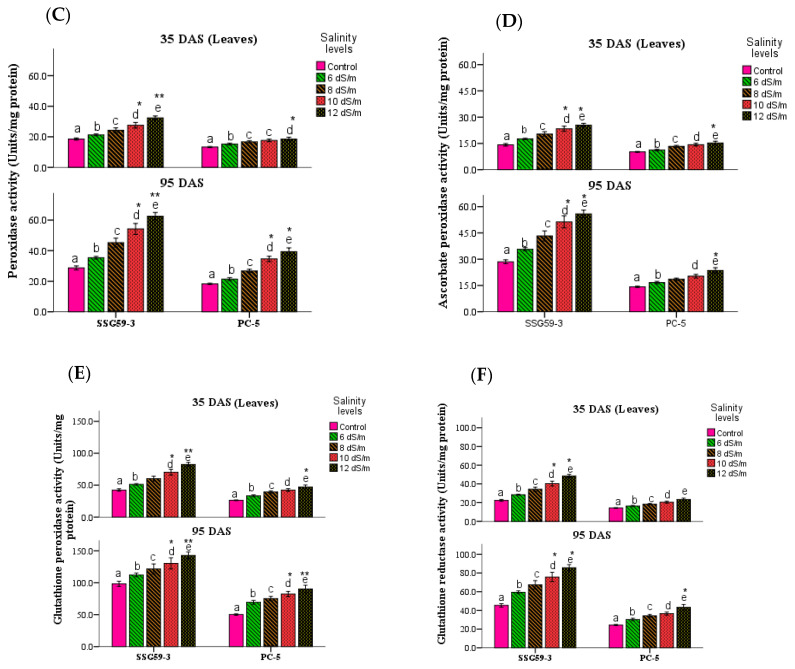
Effect of salt stress on superoxide dismutase (SOD, **A**), catalase (CAT, **B**), peroxidase (POX, **C**), ascorbate peroxidase (APX, **D**), glutathione peroxidase (GPX, **E**), and glutathione reductase (GR, **F**) of sorghum genotypes at 35 and 95 DAS. All values are the means ± SD of three biological replicates. Asterisks represent significant (*) and highly significant (**) differences among different treatments. ^a–e^ Values with different superscripts in the same row are significantly different at *p* < 0.05.

Mild or severe salinity stress increased SOD activity in sorghum leaves in a concentration-dependent manner (Figure 6A). At 35 DAS, the percent increase in SOD activity in leaves was at its maximum in SSG 59-3 (51%); therefore, it could be considered as a salt-tolerant genotype at 100 mM NaCl. In contrast, the increase was less in PC-5 (19%), indicating that it is a salt-susceptible genotype. The SOD activity was more pronounced at higher salt concentrations, i.e., at 120 mM NaCl, with an increase of 62% in SSG 59-3 and 32% in PC-5 leaves. A similar increase in SOD activity was observed at 95 DAS, but the percent increase was less pronounced in 35 DAS than 95 DAS. Differential responses of CAT activity (Units mg^−1^ protein) are shown in Figure 6B. At 35 DAS, a significant increase in CAT activity was observed in SSG 59-3 (46%) leaves, as well as a slight increase in PC-5 (22%), at 100 mM NaCl. At 120 mM NaCl, the percent increase in CAT activity was higher in SSG 59-3 (62%) as compared to PC-5 (32%). At 95 DAS, CAT activity had a maximum increase in SSG 59-3 and a minimum increase in PC-5. An increase in POX activity was observed in both sorghum genotypes (Figure 6C). In leaves at 35 DAS, the increase in POX activity in SSG 59-3 was 58% and 62% at 100 mM NaCl and 120 mM NaCl, whereas PC-5 had slight increase (22% and 29%). A similar trend in POX activity was observed at 35 DAS with values approaching about 4.4 fold in genotype SSG 59-3, while they approached 2.9 fold in PC-5 under saline conditions. At 100 mM NaCl, the maximum increase in POX activity was found in the salt-tolerant genotype SSG 59-3 (26%) while it was less in PC-5 (18%) at 35 DAS. A significant increase in specific activity was observed at a higher salt concentration (120 mM NaCl), from 32% in SSG 59-3 to 22% in PC-5. Similar results were noticed at 95 DAS. At 35 DAS, APX activity increased significantly in SSG 59-3 (55%), whereas in PC-5, the increase (26%) in APX activity was slightly less at 100 mM NaCl (Figure 6D). Further increases in salt concentration, i.e., at 120 mM, NaCl enhanced the APX activity; this was more pronounced in SSG 59-3 (65%) and less pronounced in PC-5 (23%). The same trend was observed at 95 DAS, but overall, there was more APX activity at 95 DAS than at 35 DAS.

Differential responses of GPX activity (Units mg^−1^ protein) showed a significant increase (Figure 6E). At 35 DAS, a significant increase in GPX activity was observed in the leaves of the salt-tolerant genotype SSG 59-3 (45%), with a slight increase in salt-sensitive genotype PC-5 (24%) at 100 mM NaCl. At 120 mM NaCl, the GPX activity was higher in SSG 59-3 (58%) as compared to PC-5 (33%). At 95 DAS, a similar increasing trend in GPX activity was observed with a maximum in SSG59-3 and a minimum in PC-5. Salt stress increased the GR activity in both the tolerant and susceptible sorghum genotypes (Figure 6F). However, the increase was found to be higher in tolerant genotypes. In the SSG 59-3 genotype, GR activity increased by 48% and 55% compared to PC-5, where the percent increase was 22% and 33% at 100 mM NaCl and 120 mM NaCl, respectively, in the vegetative stage. Among the studied genotypes, PC-5 showed the lower, while SSG 59-3 had the higher GR activity under salinity conditions.

### 3.5. Non-Enzymatic Oxidants

**Figure 7:**
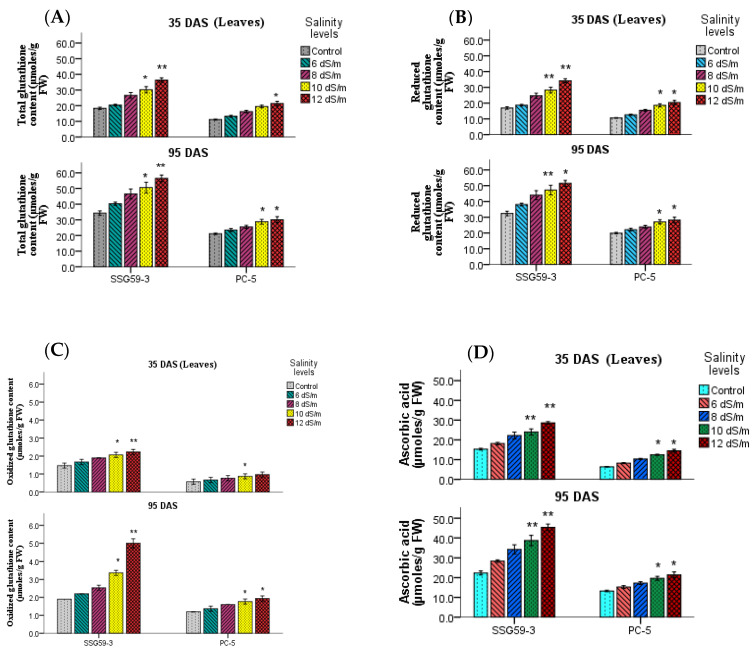
Effect of salt stress on total glutathione (**A**), reduced glutathione (GSH, **B**), oxidized glutathione (GSSG, **C**) and ascorbic acid (ASC, **D**) of sorghum genotypes at 35 and 95 DAS. All values are the means ± SD of three biological replicates. Asterisks represent significant (*) and highly significant (**) differences among different treatments.

Salt stress resulted in a significant increase in total glutathione content in leaves at both stages (Figure 7A). At 100 mM, the total glutathione content in SSG 59-3 was 54% compared to PC-5, where the percent increase was only 21% compared to their controls. A higher concentration of Na^+^ ions had a pronounced effect on total glutathione content, which further increased the glutathione content by 69% in SSG 59-3 and 32% in PC-5 compared to their respective controls. A higher concentration of Na^+^ ions had a pronounced effect on total glutathione content, further increasing the GSH content (Figure 7B) by 56% in SSG 59-3 and 31% in PC-5. In oxidized glutathione (GSSG), the percent increase was more in the case of SSG 59-3 (52%) than PC-5 (27%) (Figure 7C). There was a significant accumulation in ascorbic acid (ASC) under salinity stress (Figure 7D). At 35 DAS, ASC content increased by 52.51% in SSG 59-3 compared to PC-5, where the percent increase was only 23% at 100 mM NaCl.

### 3.6. Compatible Solutes

**Figure 8:**
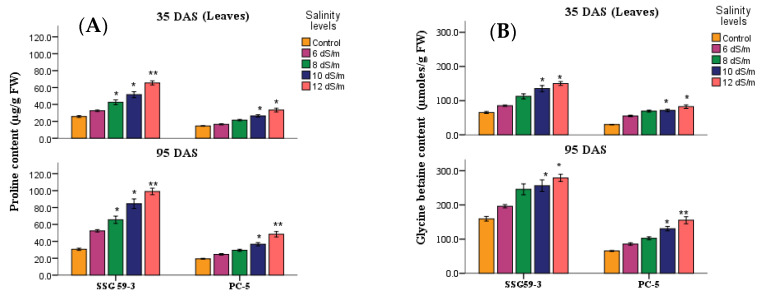
Effect of salt stress on proline (Pro, **A**), and glycine betaine (GB, **B**) of sorghum genotypes at 35 and 95 DAS. All values are the means ± SD of three biological replicates. Asterisks represent significant (*) and highly significant (**) differences among different treatments.

The accumulation of compatible solutes viz. proline (Figure 8A) and glycine betaine (Figure 8B) increased considerably in leaves of sorghum genotypes under salt stress. For proline, the percent increase was at its maximum in SSG 59-3 (50%), while PC-5 had a minimum of 23%. For glycine betaine, the percent increase was at its maximum in SSG 59-3 (58%), while PC-5 increased by 23% at 100 mM.

### 3.7. Oxidative Stress Markers

**Figure 9:**
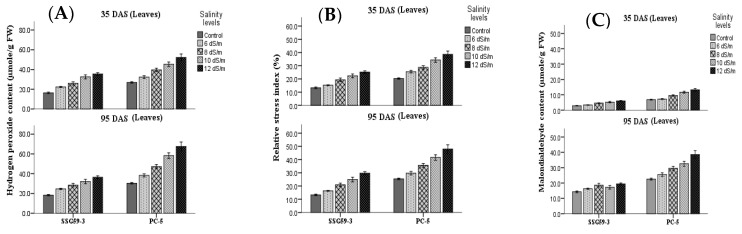
Effect of salt stress on hydrogen peroxide (H_2_O_2_, **A**), relatives stress index (RSI, **B**), and malondialdehyde (MDA, **C**) of sorghum genotypes at 35 and 95 DAS. All values are the means ± SD of three biological replicates.

Salt stress also leads to a higher accumulation of ROS, which disturb cellular redox homeostasis and result in oxidative damage. H_2_O_2_ content and markers of oxidative damage of cell membranes, i.e., the relatives stress index (RSI) and malondialdehyde (MDA) were measured (Figure 9). An increase in the NaCl concentration resulted in a significant increase in the accumulation of H_2_O_2_ content, more particularly in leaves than roots at the *p* < 0.05 level (Figure 9A). At 35 DAS, H_2_O_2_ content increased significantly in genotype PC-5 (42%), whereas its level declined in SSG 59-3 (16%) at 100 mM NaCl. Further increases in salt concentration, i.e., at 120 mM NaCl, enhanced the H_2_O_2_ content, more in PC-5 (51%) and less in SSG 59-3 (23%). The same trend was observed at 95 DAS, but overall, the H_2_O_2_ content was more at 95 DAS than 35 DAS. Following the trend of H_2_O_2_, RSI (Figure 9B) and MDA (Figure 9C) also increased in leaves, particularly at 120 mM NaCl in PC-5. Thus, oxidative stress markers showed that leaf tissues were significantly affected by the salinity-induced oxidative stress. SSG 59-3 accumulated fewer stress markers due to oxidative damage.

### 3.8. Transcriptome Assembly and Statistics

**Figure 10:**
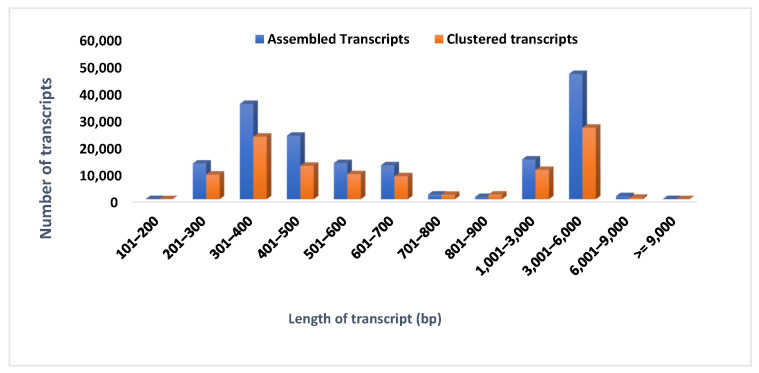
Sequence characterization of assembled and clustered transcripts of sorghum.

For sequence generation, Illumina paired-end transcriptome sequencing was utilized to generate approximately ~323.49 million reads from two sorghum cultivars in two different environmental conditions (control/stressed plants), the downstream analysis (Supporting Table S1). About ~92.14% of clean reads were obtained after the quality control check (Q ≥ 30). These high-quality reads were de novo assembled into the transcripts using Trinity for *Sorghum bicolor*. The transcriptome coverage efficiency was evaluated by comparing the unique gene sequences in close proximity with the available transcriptome in de novo sequencing [35,36]. The assembly of these high-quality clean reads gave 143,264 transcripts among all the salinity treatments with an N50 of 1649 bp and a contig length of 1145 bp. The short stretches of gene sequences might have lacked a well-characterized protein domain, thereby failing to show sequence matches, which resulted in false detection rates (Figure 10). The contigs >300 bp in length were excluded from the transcript assembly. Altogether, from control and salt-treated (6, 8, 10, and 12 dSm^−1^) transcriptome libraries, a total of 90.37 million, 85.95 million, 83.56 million, 80.74 million, and 78.36 million raw reads were generated, respectively, for each group (~92.12%) and further used for the downstream analysis (Table 3).

### 3.9. Differential Gene Expression Analysis

**Figure 11:**
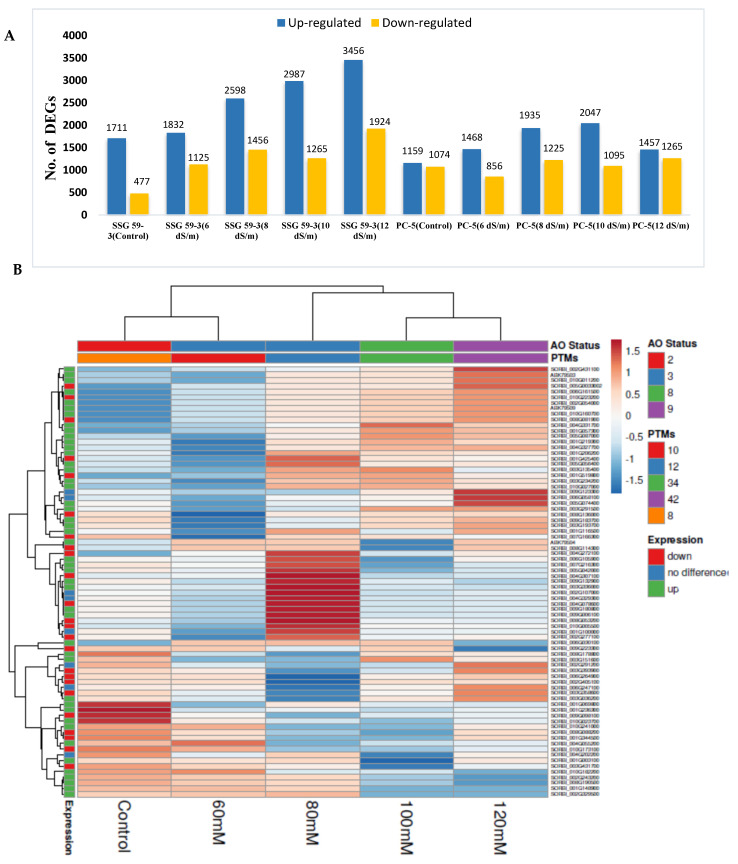
An (**A**) overview of DEGs in different combinations; (**B**) heat map and clustering of the top 50 salinity-responsive DEGs in control and stressed shoot tissues of SSG 59-3 (salt-tolerant) and PC-5 (salt-sensitive) sorghum genotypes using Clustvis (https://biit.cs.ut.ee/clustvis/, accessed on 19 June 2021).

In total, 32,412 DEGs were detected, of which 20,650 were upregulated and 11,762 were downregulated. By comparing the number of DEGs between control and salt-treated tissues of both tolerant and susceptible genotypes, the following several DEGs were obtained: SSG 59-3 (1284 upregulated; 624 downregulated) and PC-5 (806 upregulated; 515 downregulated) (Figure 11A). Further experimentation included analyzing the expression profiling of the DEGs regulated under both control and salt stress treatments across both cultivars. The heat map of the top 50 salinity-responsive DEGs, scaled on FPKM expression values, demonstrated that all the DEGs exhibited differential expressions in the samples for control and stressed tissues, indicating that the key response mechanism varied between the tolerant (SSG 59-3) and susceptible genotype (PC-5). Thus, based on their adaptability behavior and salt treatments, the genotypes were classified into similar groups, each exhibiting different gene expression patterns for the control and salt-treated plants (Figure 11B).

### 3.10. Comparative Co-Expression Analysis of DEGs

**Figure 12:**
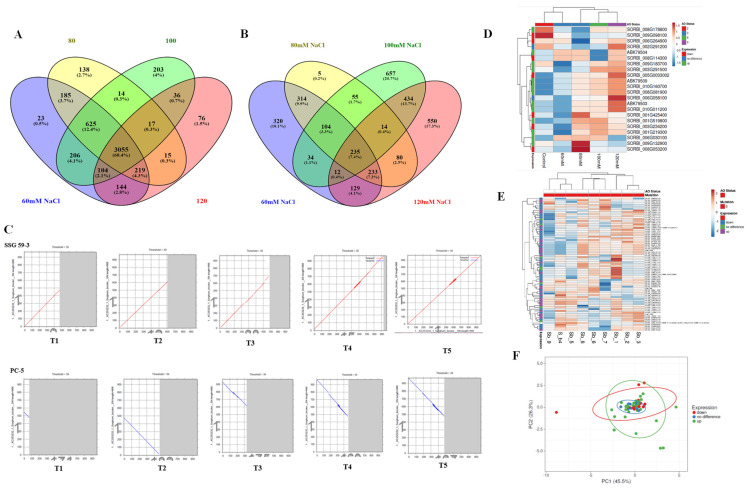
Number and grouping of DEGs. (**A**,**B**) Venn diagram analysis of DEGs identified in sorghum genotypes under the five experimental comparisons. Principal component analysis (PCA) showing clusters under different salinity treatments in sorghum genotypes. (**C**) Overview of DEGs SSG59-3 and PC-5, (**D**,**E**) heatmap representing top 22 up- and downregulated transcripts, (**F**) principal component analysis of the clusters as per the treatment.

A comparative co-expression transcriptome analysis was evaluated to delineate the DEGs underlying the salinity tolerance mechanism in sorghum genotypes by comparing the tolerant genotype (SSG 59-3) with susceptible genotype (PC-5) (Figure 12A–D). The Venn diagram generated using Venny 2.1.0 (https://bioinfogp.cnb.csic.es/tools/venny/, accessed on 6 July 2021) revealed 625 transcripts, (12.4%), which were commonly up-regulated at 6, 8 and 12 dS/m, 3055 (60.4%) transcripts between 6, 8, 10 and 12 dS/m, and 76 transcripts (1.5%) were only at 12 dS/m in SSG 59-3 (Figure 12A,D), while in PC-5, 235 (7.4%) transcripts between 6, 8, and 12 dS/m, 104 (3.3%) transcripts between 6, 8, and 10 dS/m, 657 (20.7%) transcripts exclusively at 10 dS/m, and 550 (17.3%) transcripts exclusively at 12 dS/m were found (Figure 12D). It also presents a modular architecture of the biosynthesis pathways of stress-responsive genes (Figure 12E) among different salt treatments. Each gene may be viewed as consisting of the core part and its extensions. The core part represents the KEGG module.

Furthermore, the bottom extension of essential amino acids appears to be most divergent, containing multiple pathways for metabolite biosynthesis and multiple gene sets for antioxidant component biosynthesis. In the heatmap, red and green colored bands recognize higher gene expressions and antioxidant status. At the same time, the blue and purple colors represent the low gene expression level and antioxidant status, respectively. The principal component analysis (PCA) showed that the first two principal components (PCs) explicated most of the variance (45.50%), and the cultivars were grouped into a similar cluster as per salt treatment (Figure 12F).

### 3.11. Functional Classification of DEGs, GO Analysis, and Similarity Search

**Figure 13:**
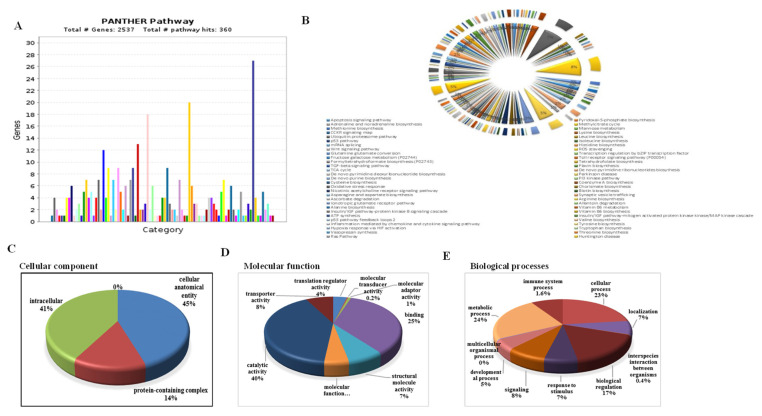
GO terms: (**A**) Gene Ontology classification of metabolic pathways, (**B**) frequency of highly abundant GO terms under the molecular function, biological process, and cellular component categories in *Sorghum bicolor*, (**C**) GO classification based on the cellular level, (**D**) GO Ontology classification based on molecular function, (**E**) GO classification based on biological processes.

Based on functional enrichment of GO, the annotated transcripts were categorized into three different classes viz. molecular function (MF), biological processes (BP), and cellular component (CC), respectively. GO hits 2537 transcripts, covering 360 pathways (Figure 13A). The most abundant terms from each ontology class are represented in a donut chart (Figure 13B). Terms such as an integral membrane and ion transporter category were highly expressed in the salt-tolerant SSG 59-3 genotype (Figure 13C). The classification based on the cellular component revealed that the majority of DEGs (3 classes) were present in the intracellular region (41%), followed by cellular and anatomical activity (45%), and the protein-containing complex (14%), while in PC-5, four categories were observed that were present in the intracellular region (43%) followed by the biosynthetic process (23%), cellular and anatomical activity (21%), and the protein-containing complex (11%). For the MF category, GO terms such as catalytic activity, signaling transduction pathways, transmembrane transporter activity, receptor kinase activity, integral membrane components, and ATP binding comprised several novel transcripts in the SSG 59-3 genotype (Figure 13D). Classification based on molecular function showed that the majority of the DEGs were classified into eight categories involved in catalytic activity (57%), binding proteins (30%), structural molecular activity (5%), translation regulator activity (3%), transported activity (3%), molecular function regulator (2%), molecular transducer activity (0.4%), and molecular adaptor activity (0.4%). Notably, in the BP category, terms including response to stimuli, cellular processes, and metabolic functions constituted highly represented transcripts (Figure 13E). On the basis of biological processes, the DEGs were classified into 12 categories, including those involved in cellular processes (44%), metabolic processes (35%), biological regulation (7%), localization (7%), response to stimulus (6%), biological phases (1.8%), reproductive processes (1.2%), signaling (1%), developmental processes (0.6%), reproduction (0.5%), multicellular organismal processes (0.4%), and immune system processes (0.4%).

**Figure 14:**
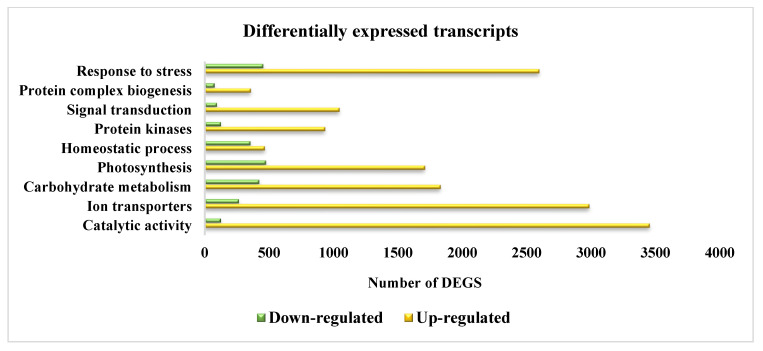
Most highly represented pathways in *Sorghum bicolor*.

These 15,756 unique transcripts were functionally annotated using the KAAS system, and antioxidative metabolites biosynthesis was shown to have a lower number of homologous transcripts. The most abundant annotated transcripts were detected for catalytic activity (3456 transcripts), response to stress (2598 transcripts), and ion transporters (2987 transcripts) (Figure 14). The KEGG enrichment for the most common terms is given in Supporting Figure S1.

### 3.12. DEGs Network Analysis

**Figure 15:**
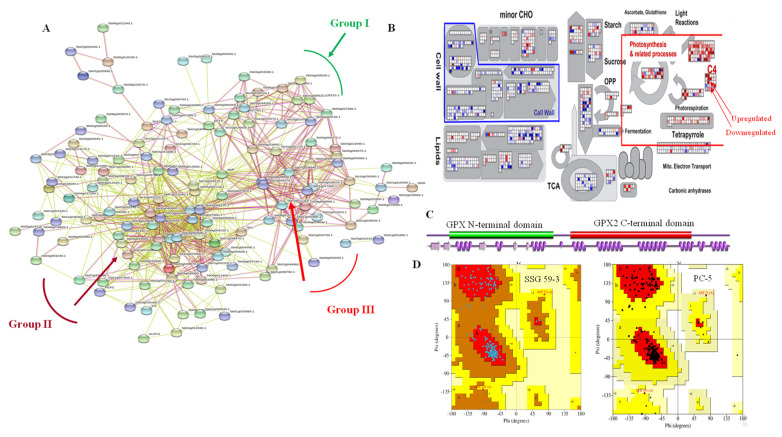
Network analysis: (**A**) STRING-based interaction network analysis of DEGs, (**B**) MapMan pathway analysis, (**C**) 3-d structure of glutathione peroxidase gene using PDBsum, (**D**) Ramachandran plot of amino acid residues in salt-tolerant and susceptible genotype.

Out of the selected candidate genes that played an essential role under salinity and were validated through qPCR analysis, there were substantial interactions among the DEGs analyzed utilizing the STRING network analysis database, with a confidence level of >0.5 (Figure 15). In our study, *NCED3* (XP_004508176.1), a critical ABA synthesis gene in response to abiotic stresses, interacted with proteins such as CEVI57-like proteinase inhibitor thaumatin-like protein and also played an essential role in signal transduction pathways (Group I; Figure 15A). Likewise, the genes that play a significant role in epigenetic responses and cell wall remodeling under different abiotic stresses, such as antioxidative enzymes, transcription factors, protein kinases, methyltransferase, and ion transporters, also interact with other proteins that play similar roles in salinity (Group II; Figure 15A). Network analysis of important TFs, including *NAC1*, *MYB*, and *ERF*, exhibited complex interactions with several other salt-responsive proteins whose expressions were upregulated under elevated salt stress, such as *LEA*, *WRKY*, *bHLH92*, zinc finger protein, and *E3 ubiquitin-protein ligase* (Group III; Figure 15A). Other important genes were found to be upregulated under elevated salinity, including such aspects as proline-rich extensin-like proteins, structural cell wall components, signaling, calcium-binding protein CML18, threonine aldolase-like, and dihydro flavonol-4-reductase. MapMan (v.3.5.1) analysis revealed that during high salinity, there was upregulation of photosynthesis and related genes (such as ribulose bisphosphate carboxylase, PEP-utilizing enzyme family, and ATP synthase subunit beta) in the salt-tolerant genotype (Figure 15B). Using the PDBsum (https://www.uniprot.org/database/DB-0119/19.06.2021, accessed on 19 June 2021), a pictorial database of antioxidative enzyme glutathione peroxidase (GPX) is depicted in Figure 15C, which represents the schematic overview of its different domains and their interactions. The Ramachandran plot was also generated using PDBsum, representing the active sites, protein residues, covalent forces in both tolerant and susceptible genotypes (Figure 15D).

### 3.13. Phylogenetic Tree, Structure and Motif Analysis of SbNAC1 TFs

**Figure 16:**
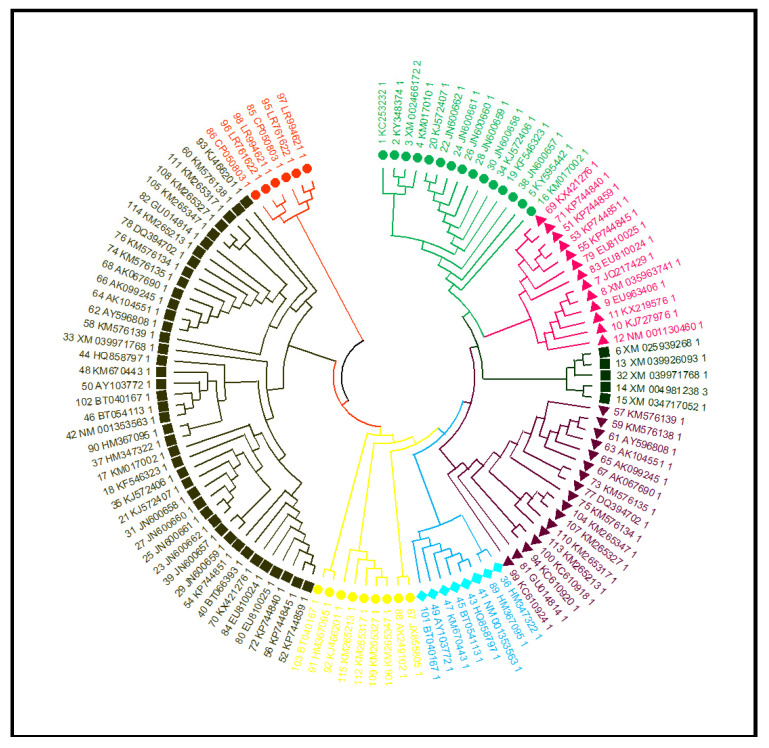
Phylogenetic tree of *NAC1* proteins from sorghum using the Mega X (ML method). The colored arcs display different groups. The green circles, pink triangles, green squares, brown triangles, blue stars, yellow circles, brown squares, and red circles represent *Sorghum bicolor*, *Oryza sativa*, *Miscanthus lutarioriparius*, *Panicum virgatum*, *Setaria viridis*, *Hordeum vulgare*, *Zea mays*, and *Digitaria exilis*, respectively.

A total of 129 amino acid sequences from related species (i.e., *Zea mays*, *Oryza sativa*, *Miscanthus lutarioriparius*, *Panicum virgatum*, *Setaria viridis*, *Hordeum vulgare*, and *Digitaria exilis*) were compared to assess their evolutionary relationships with each other. A phylogenetic tree was constructed in MEGA X software using the ML method (Figure 16). Around 129 *SbNAC1* TFs were identified from the topology of the ML tree, and these identified TFs were categorized into eight groups.

Based on the 85 amino acid sequences of the *SbNAC*1 protein, the phylogenetic tree was built using the ML method in MEGA X. The motifs and gene structures were grouped and clustered as per the topology of the phylogenetic tree (Supporting Figure S2A). The diversification of the identified gene structures plays a crucial role in the evolution of the gene family. Our results revealed that the majority of the *SbNAC*1 TF genes had no or 1–2 introns per gene, with *SbNAC*1-67 having nine introns (Supporting Figure S2B). More precisely, no introns were detected in 30 *Sb*S*NAC*1 genes (59%), while 1 or more introns were recognized in 11 (15%) and 67 *SbNAC*1 genes (13%), respectively. MEME was used for the amino acid sequence analysis and to further characterize the structure of *SbNAC*1 TFs (Supporting Figure S2C). The DNA-binding domain of *SbNAC1* TF revealed the major amino acid residues involved in the response to salinity (Supporting Figure S3).

### 3.14. DEGs Playing Role under Salinity

**Figure 17:**
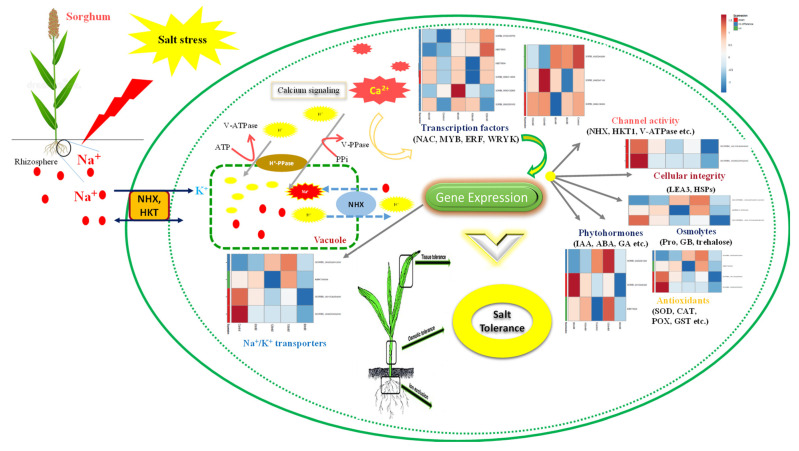
Schematic representation of the molecular mechanisms underlying salinity tolerance acquisition under different salt concentrations.

The differentially expressed genes in response to high salinity in sorghum were found to be associated with several metabolic and biological processes, including K^+^ transporter-like protein HAK/KUP transporter, sodium proton antiporters such as *NHX1*, responses to oxidative stress, photosynthesis, osmotic stress, *PP2C* family protein, MAP kinases, positive and negative regulation in response to salinity, Ferredoxin-NADP reductase, protein kinase signaling cascade, calcium-binding proteins, trehalose metabolism, pectinesterase, stress granule assembly, and MIP/aquaporin. These DEGs are engaged in transporter functions, cellular membrane integrity, stress response, and signal transduction and have been related to salt stress rendering essential functions. The overall schematic representation of common DEGs between tolerant (SSG 59-3) and susceptible (PC-5) genotypes was studied to explain the potential role of these differentially expressed genes in sorghum under salt stress (Figure 17).

#### 3.14.1. Differentially Expressed Genes Encoding TFs

TFs encoding transcripts were differentially regulated, 103 were upregulated, and 91 were downregulated (Figure S4A). These identified TFs were related to 25 different families, which are key regulators in salinity stress, such as *NAC*, *bZIP*, *ERF*, *MYB*, *WRKY*, *C*_2_*H*_2_, and *HSF*, with 58% related to the *NAC*, *MYB*, *ERF*, and *WRKY* families. The majority of the TFs encoded by the TFs encoding transcripts were upregulated in the salt-tolerant genotype (Supporting Figure S4B).

#### 3.14.2. Ion Transporters

Under high salinity conditions, signal perception is mediated via calcium-dependent protein kinases (CDPK24; SORBI_003G291500), which helps in the signal relay mechanism, and the fold expression was 3.2 times induced in tolerant genotype. Na^+^ and K^+^ proton transporters such as V-ATPase (SORBI_010G027000), HATPase C domain-containing protein (SORBI_002G243200), plasma membrane ATPase (SORBI_008G190500), H(^+^)-exporting diphosphatase (SORBI_004G068300), and vacuolar proton pump subunit B (SORBI_004G094700), which are crucial for Na^+^/K^+^ transport and the maintaining of ion homeostasis, were upregulated in the tolerant genotype.

#### 3.14.3. Photosynthesis

High salinity drastically affected the photosynthetic efficiency of plants due to a reduction in accessory photosynthetic pigments, a reduction in the unsaturation index, and ultimately, a reduction in quantum yield. Photosynthetic pigments such as phycobilins and chlorophyll were also degraded under high salinity. Another enzyme, rubisco decarboxylase (ABK79504), involved in biomass accumulation, was upregulated under salt stress. Fascinatingly, in sorghum, the gene expression of the photosynthetic encoding enzymes, i.e., phosphoenolpyruvate carboxylase (SORBI_010G160700), the ribulose bisphosphate carboxylase large chain (ABK79504), phosphopyruvate hydratase (SORBI_010G027000), malate dehydrogenase (SORBI_001G219300), malic enzyme (SORBI_003G036200), rubisco activase beta isoform, an apoprotein A2 (psaB), photosystem I P700, and the photosystem II CP43 reaction center protein, were highly upregulated in response to high salinity.

#### 3.14.4. Oxidative Stress

In the present study, it was found that DEGs, including catalase (SORBI_04g001130), L-ascorbate peroxidase (SORBI_002G431100), superoxide dismutase (SORBI_009G093200), glutathione reductase (SORBI_004G341200), peroxidases (SORBI_009G055300), lipoxygenase (SORBI_001G125900), serine/threonine-protein phosphatase 2A (SORBI_007G024700), glutaredoxin-dependent peroxiredoxin (SORBI_004G076000), lambda glutathione transferase (SORBI_001G412800), thioredoxin domain-containing protein (SORBI_10g026630), and alkaline alpha-galactosidase, were upregulated with increasing salt levels in response to oxidative stress.

#### 3.14.5. Compatible Solutes

The transcripts betaine aldehyde dehydrogenase 2 (SORBI_07g020650), Δ-1-pyrroline-5-carboxylate synthase (SORBI_110430201), and trehalose 6-phosphate phosphatase (SORBI_09g025660) were found to be upregulated, indicating the accumulation of glycine betaine, proline, and trehalose with increasing salt concentrations.

### 3.15. Validation of the Differential Gene Expression Analysis

**Figure 18:**
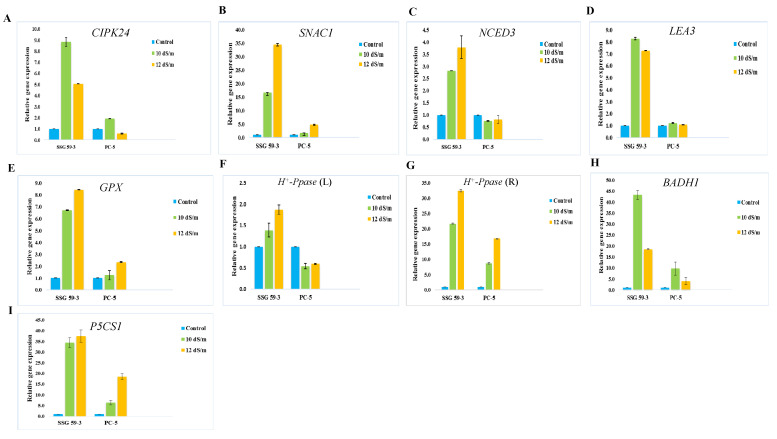
The expression levels (**A**–**I**) of stress-related marker genes of 10 selected DEGs based on qPCR. The *PP2A* gene was used as the reference gene/internal control. Data are shown as mean ± S.D. (n = 3).

The reliability and the validation of sequencing results obtained from the Illumina Hiseq-2500 platform were confirmed by qPCR studies. The qPCR expression analysis was conducted to identify and confirm if the pathways of the selected candidate genes were involved in providing tolerance to high salinity in sorghum genotypes. A similar set of samples was prepared under the same experimental conditions as those used for RNA-seq. The DEGs, selected based on their differential roles under salt stress, were subjected to qPCR analysis. Of these selected genes, the relative expression levels of CBL-interacting serine-threonine protein kinase 24 (*CIPK24*), glutathione peroxidase (*GPX*), late embryogenesis abundant proteins (*LEA3*), stress-induced *NAC* protein 1 (*SNAC1*), 9-cis-epoxycarotenoid dioxygenase (*NCED3*), vacuolar H^+^-pyrophosphatase (*H^+^-PPase*), betaine aldehyde dehydrogenase (*BADH1*), Δ1-pyrroline-5-carboxylate synthetase 1 (*P5CS1*), actin (*Act*), and protein phosphatase 2A (*PP2A*) were analyzed in two sorghum genotypes, viz. SSG 59-3 (salt-tolerant) and PC-5 (salt-susceptible), under two different salt concentrations, i.e., at 10 and 12 dS m^−1^ (Figure 18). *PP2A* was used as reference gene/internal control for data normalization. These two different salt concentrations were used for gene expression study, as the significant differences were not observed at 6 and 8 dS m^−1^.

RNA integrity was assessed by horizontal agarose (1.5%) gel electrophoresis (Supporting Figure S5A,B), and single-band samples specified the gene of interest as well as single melting curve peaks (Supporting Figure S5C–J). The relative expression of these genes was upregulated in tolerant (SSG 59-3) and downregulated in sensitive (PC-5) genotypes under salt stress (Figure 18A–I). Genes such as *CIPK24*, *LEA3*, and *BADHI* were highly upregulated at 10 dS/m, while the genes *SNAC1*, *NCED3*, *GPX*, and *P5CS1* were more expressed at 12 dS/m. *H^+^-PPase* transporter was highly upregulated in roots as compared to leaves in salt-tolerant genotypes. All ten genes followed similar expression trends (up- or downregulation) in both the RNA-seq analysis and qPCR.

The stability values for each candidate gene varied from one experimental set to another experimental set. The stability ranking of the candidate gene orders from three sample sets was evaluated separately to recognize the most stable genes by means of three different statistical tools, NormFinder, the ΔCt method, and geNorm. The expression stability rankings for the selected candidate genes, obtained via the geNorm finder, showed that the lowest M values were observed for the *H^+^-PPase*, *PP2A*, and *NCED3* genes (*M* = 0.76), which were identified as having the most stable expression in all sample groups (Supporting Figure S6A). In contrast, the M values for *LEA, BADH1,* and *P5CS1* were significantly higher. The cut-off value was set at a value of 1.5; M values lower than 1.5 indicated higher stability, and values higher than 1.5 indicated lower stability. The direction of the arrow displays the least and most stable candidate gene expressions. The least stable genes are listed on the left side, and the most stable genes are listed on the right side. *PP2A* was used as the most stable reference gene/internal control for the salt stress treatments in the present study. The criteria used for NormFinder (Supporting Figure S6B) rankings were slightly different from those used for geNorm rankings (Figure 15A). Both statistical tools ranked the *H^+^-PPase* and *PP2A* genes as the most stably expressed genes in control and stressed plants, while the *BADH1* and *P5CS1* genes showed the least stability based on M-values (< 1.5). The comprehensive ranking (Supporting Figure S6C) of the candidate stress-responsive genes for all three different experimental samples were highly consistent with the results of NormFinder and geNorm. The comprehensive ranking showed that *SNAC1*, *LEA3*, *P5CS1,* and *BADH1* were the least stable genes in all samples under salt stress and were highly upregulated under stress conditions.

## 4. Discussion

In the current climate change scenario, environmental stresses, specifically salt stress, constrain crop plant growth, physiology, and productivity by accumulating toxic ions such as Na^+^ and Cl^−^ [35,36,37]. The phenotypic and morpho-physiological characteristics of sorghum were investigated at different salt concentrations to better understand the salt stress tolerance mechanisms. The present study sought to delineate the underlying molecular mechanisms of salinity tolerance in well-characterized sorghum cultivars viz. SSG 59-3 (tolerant) and PC-5 (susceptible). Phenotypic and morpho-physiological traits, such as the membrane injury index, the RWC, the chlorophyll content, and the ionic distribution of Na^+^ and K^+^ ions, of the susceptible genotype were more affected than those of the tolerant genotype under salt stress (Figure 4) [5].

Seedling growth is a critical stage for the establishment of plant populations under saline conditions. Sorghum displays a significant intra-specific difference in salinity tolerance. Seedling growth parameters viz. fresh and dry weight and root and shoot length (Figure 3) decreased significantly with increasing salt concentrations. The degree of reduction varied with the salinity levels and genotypes (*p* ≤ 0.05); more reduction in the sensitive genotype, PC-5, than the tolerant genotype, SSG 59-3, was observed. Salinity primarily reduces soil solutions’ osmotic ability to impede water intake by seed, thereby affecting the seed germination rate [38], and minimizes the ease with which the seeds absorb water or the toxicity of Na^+^ and Cl^−^ ions, even in the case of salt-tolerant plants [13].

Salt stress severely hampers crop plants’ physiological parameters, which could be due to decreased leaf expansion, premature leaf senescence, impaired photosynthetic machinery, and changes in the structure of proteins and pigments. Tolerant genotype (SSG 59-3) had higher chlorophyll content and RWC but lower relative stress index (RSI)/electrolyte leakage than sensitive genotype (PC-5) (Figure 4). Higher RWC could help the tolerant genotypes to perform physico-biochemical processes more efficiently under stress conditions than susceptible sorghum genotypes. Earlier studies have shown that these morpho-physiological characteristics could be used as salt tolerance biomarkers. This is owing to the more remarkable ability and speed of water absorption in tolerant plants under adversity to prevent tissue dehydration. The lower RSI ratio in the SSG 59-3 genotype shows their ability to protect the cellular membrane network against uncontrolled RSI from salt stress [39]. The retention of higher chlorophyll is necessary because a higher concentration of chlorophyll pigments effectively reduces the photo-inhibition of the photosynthetic apparatus in mesophyll cells and reduces carbohydrate degradation required for seed growth. A gradual reduction in chlorophyll in susceptible genotypes under salt stress may be associated with ROS generation, leading to the oxidation of chlorophyll, a reduction in photosynthetic efficiency, and the degradation of other chloroplastic pigments coupled with several pigment-protein complexes [5]. Furthermore, excess Na^+^ and Cl^–^ ions damage the cellular membrane network, which can also be linked to the replacement of Mg^2+^ ions.

The susceptible genotype (PC-5) exhibited greater Na^+^/K^+^ ratios than the tolerant genotype (SSG 59-3), indicating that the concentration of Na^+^ and K^+^ ions in plant cells is essential for tolerance [40]. The concentrations of Na^+^ and K^+^ and their ratio are critical aspects in investigations of the salinity tolerance responses. Our research findings depict that the values of Na^+^ content in root were considerably higher than leaves with the increasing of salinity levels, while the K^+^ content decreased. SSG 59-3 maintained lower Na^+^ ions (Figure 5). It imported more K^+^ ions, so it had a lower Na^+^/K^+^ ratio. In comparison, PC-5 excluded fewer Na^+^ ions and had a higher Na^+^/K^+^ ratio because Na^+^ effectively competes with K^+^ for uptake in a common transport system, as shown in previous studies [34]. Ionic exchangers, cellular membrane integral and carrier proteins, antiporters, toxic ion exclusions, and a compartmentation strategy that prevents acute Na^+^ cytotoxicity at high salt concentrations, are adapted by the salt-tolerant genotype (SSG 59-3) to maintain cellular ion homeostasis [41].

In the present study, attempts were made to establish a correlation between antioxidative defense mechanisms and salinity-induced changes in the leaves and roots of sorghum genotypes at two growth stages, viz. the vegetative stage and physiological maturity. Under saline conditions, plants have to activate different physiological and biochemical mechanisms in order to cope with the resulting stress [42,43]. During oxidative stress, the excess production of ROS is scavenged by a complex enzymatic antioxidative system that controls ROS production and ultimately protects the plant against oxidative damage [44]. This salinity-induced defense mechanism is differential and primarily dependent on differential antioxidant enzymes, salinity extent, and exposure time [45]. SOD is the most effective intracellular enzymatic antioxidant; it is ubiquitous in all aerobic organisms and subcellular compartments that are prone to ROS-mediated oxidative stress. Lee et al. [46] reported that transgenic tobacco plants overexpressing Cu/Zn-SOD showed tolerance to salt and water stresses. The induction of catalase activity was reported with the accumulation of H_2_O_2_ and is seemingly consistent with this enzyme’s role in scavenging-enhanced H_2_O_2_ levels. Greater salinity-induced stimulation upon POX activity in tolerant genotypes than in sensitive ones suggested their possible role in efficiently removing H_2_O_2_ in tolerant genotypes. Bhattacharjee and Mukherjee [47] reported a gradual decline in POX activity, increasing heat shock and salinity stress in *A. lividus*. Enhanced peroxidase activity under various stresses was linked to protection from oxidative damage, lignification, and cross-linking of the cell wall. APX and GPX are specific enzymes that scavenge chloroplastic H_2_O_2_ using ascorbate as an electron donor in the first step of the ascorbate–glutathione cycle and are considered to be the essential plant peroxidases in H_2_O_2_ detoxification [48]. The enhanced activity of APX and GPX, concomitant with an enhanced ascorbic acid and glutathione content, may help to quench ROS. GR is essential to recycle GSH in the ascorbate–glutathione cycle in NADPH-dependent reactions. Similarly, higher induction in GR activity in tolerant varieties than susceptible varieties was reported in *Macrotyloma uniflorum* and chickpea. Similar results were also reported in maize and wheat, although they differed in terms of salt tolerance.

Non-enzymatic antioxidants include ascorbic acid, phenolics, glutathione, and carotenoids. Glutathione is a major non-protein thiol in plants, which plays a pivotal role in protecting plants from environmental stress. The diminished ascorbate pool under various stresses may be due to changes in the glutathione pool that have been implicated in the recycling of ascorbate, or failure to maintain ascorbate levels, indicating an overall decline in the capacity to withstand oxidative stress [49]. GSH levels increased in response to high salinity, particularly in roots at the level of organ specificity. This could be ascribed to the increased demand and metabolism of sulfur under stress for antioxidants’ biosynthesis, such as GSH [50]. The increased absorption of sulfate affects GSH levels in tissues under high salinity. The increased content of ASA and GSH, accompanied by the reduced ASA and GSH redox status, indicated the crucial role of the ASA-GSH cycle for scavenging ROS.

Plants maintain their cell turgor and osmoregulatory mechanisms by accumulating compatible solutes such as proline, glycine betaine (GB), polyamines, and proteins [51] under different abiotic stresses that decreased the cytoplasmic osmotic potential, enabling water absorption. Proline is considered to be the only osmolyte that has been shown to scavenge singlet oxygen and free radicals, including hydroxyl ions, and stabilizes proteins, DNA, as well as the membrane [52,53]. Glycine betaine (GB) is one of the osmoregulatory solutes that naturally accumulate in plants [54]. GB can also protect the O_2_-evolving machinery of chloroplasts when exposed to high NaCl concentrations [55]. Saneoka et al. [56] reported that the salt-induced accumulation of betaine and BADH mRNA coincides with ABA in sorghum. The accumulation of soluble carbohydrates increases resistance against various stresses [57]. Total soluble carbohydrates act as osmolytes inside the cell. The imposition of water or salt stress in sorghum was demonstrated to be accompanied by an increase in the sugar levels of embryos, which may help in osmoregulation under stress conditions.

H_2_O_2_ is a natural plant toxic cellular metabolite; it is toxic only at high concentrations and causes thylakoid degradation. Higher accumulation of H_2_O_2_ in susceptible genotypes might be related to higher antioxidant activity and higher proline total soluble sugar content. Forghani et al. [58] observed that H_2_O_2_ content was about 54% more salt-stressed than control plants. The relative stress index (RSI), in terms of leakage of electrolytes, increased significantly under stress conditions. Increased electrolyte leakage from tissues is usually an expression of modification in the physical properties of cell membranes. Malondialdehyde (MDA) is one of the final products of the peroxidation of unsaturated fatty acids in phospholipids and is responsible for cell membrane damage. Higher production of MDA content, induced by salt stress, was reported in the present work, which agrees with those reported by several workers [5]. Salt stress-induced membrane lipid peroxidation results in membrane fluidity, leading to enhanced electrolytic leakage in wild-type and transgenic lines in sorghum [59]. During stress conditions, the total polyamine concentration was enhanced. The increased concentration of different polyamines in leaf tissue of sorghum plants suggested their potential role in ROS scavenging.

In recent years, high-throughput techniques have provided novel ways to explore the complex network of salinity response to identify key elements for stress tolerance acquisition [60]. Understanding the tolerance mechanism at the molecular level would aid in the development of salt-resistant lines in sorghum using molecular genetics approaches. Following previous studies in other crops, a comparative transcriptome analysis was conducted among the tolerant and susceptible genotypes to uncover transcriptional alterations under saline conditions [61]. Several DEGs/transcripts were identified in sorghum, and their sequence analysis revealed a genotype/treatment-specific response [62,63]. Under saline conditions, the differentially expressed transcripts engaged in different molecular, cellular, metabolic, and biological processes were found to be altered in tolerant and sensitive genotypes [64].

*Sorghum bicolor* is a high biomass-yielding crop, and is moderately drought tolerant, with broader ecological functions [3,4]. A thorough perusal of the literature showed that the lack of well-maintained work on salinity induces physio-biochemical and molecular responses of sorghum under salt stress. This is the first time a report of the transcriptome analysis of sorghum at higher salt concentrations for functional categorization of DEGs has been published. Approximately 323.49 million reads were generated using Illumina paired-end transcriptome sequencing, with 92.14% of the top reads having a Phred score of Q ≥ 30. In comparison to other EST sequencing methods, RNA-Seq offers assembled and annotated high quality reads, as evidenced in a group of plant studies covering, among others, pearl millet, sugar beet, *Halogeton glomeratus*, *Suaeda salsa*, *Spartina spp*, and *Withania Somnifera*. Normalization of the sequencing library significantly enhances the number of annotated transcripts and minimizes oversampling of abundant transcripts [65]. The fold change for a particular transcript was calculated using DESeq normalized expression values. The genes with significant differences were considered to be differently expressed between treated and control samples. Comparing with earlier findings in non-model plant species, the fraction of first annotated transcripts (61.36%) in sorghum are within the range as reported in *Spartina* and *Cicer* (67.8%), *Amaranthus* (81.9%) [35], and US Ginseng root (68.1%). Similarly, compared to the 32% rate of unique transcripts obtained in the present study, unique transcripts were reported at a rate of 36% in moth bean and 9% in maize.

More than 35% of DEGs of *S. alterniflora* have sequence similarity of > 92% compared to *Oryza sativa*, followed by *Sorghum bicolor*, *Zea mays*, *Arabidopsis thaliana*, and *Vitis vinifera* [66]. Similarly, in the present study, approximately 30% of the DEGs of sorghum had a similarity of greater than 85% with maize (*Zea mays*), followed by rice (*Oryza sativa*), *Miscanthus lutarioriparius*, *Panicum virgatum*, *Sorghum bicolor*, *Setaria viridis*, *Hordeum vulgare*, and *Digitaria exilis*.

Numerous biological, cellular, and molecular functions that play crucial roles in diverse pathways were uncovered through functional annotation of differentially expressed genes. The salinity tolerance mechanism is complicated with several metabolic, cellular, physiological, and molecular responses. The function of genes in conferring salt tolerance, such as protein kinases, osmolytes, ion transporters, and TFs, was previously documented [67]. Proline metabolism, *SOS1-SOS3* (salts excessively sensitive) pathways, MAPK (mitogen active protein kinase), and plant hormonal and calcium signaling are perhaps the most crucial pathways recognized for their salinity acquisition mechanisms [68]. *S. alterniflora* functional annotation revealed that 25% of ESTs belong to stress-related proteins and nucleic acid metabolism (15%); however, 13% of ESTs do not have known protein functions and are considered to be hypothetical predicted proteins [69]. In *Beta vulgaris*, leaves and roots had a differential expression of unigenes, representing several distinct mechanisms under 200 and 400 mM NaCl [70]. The enrichment analysis by GO and KEGG database server revealed DEGs for protein phosphorylation, signal transduction, and redox regulation. *S. salsa* exhibited differential expression of 77,671 unigènes (47,967 upregulated, 29,345 downregulated) in leaf and root tissues at 30 mM NaCl [71]. The KEGG pathway analysis revealed DEGs in *S. alterniflora* for the carbon metabolism, fatty acid metabolism, nitrogen compounds, and amino acid metabolism at 500 mM NaCl. Likewise, several DEGs have been found for nuclear regulatory mechanisms in sorghum, such as DNA transcription (regulation) and post-transcriptional modifications (PTMs), which indicate that salt stress activates the gene regulation network.

The qPCR gene expression analysis aimed to identify if the selected DEG networks were engaged in sorghum salinity tolerance. Previous reports suggested a correlation between qPCR and DEGs data; however, all validated genes exhibited similar expressions, which is consistent with DEG data. Still, the fold change was not nearly the same.

DEGs belonged to transmembrane transporter gave tolerance against salinity via modulating transcripts that regulate the transporter channels. Calcium signaling controlled by the calcium-transporting ATPase gene and CDPKs regulates the initial perception of stress signal. Calcium ions, on the contrary, activate protein kinase genes, which facilitate the mechanism of signal transduction, and the efflux of Ca^2+^ ions is necessary for sensory pathways under elevated salt stress [72], and was also documented in previous studies to confer salinity tolerance in chickpea and *Arabidopsis*. In tolerant genotypes with potential involvement in salinity tolerance, the transcript coding of the potassium channel genes *SKOR* and *HAK*/*KUP* (K^+^ transporter) was greatly enhanced. These genes are associated with the exclusion of Na^+^ ions, which improves the Na^+^/K^+^ ratio [73]. In tolerant genotypes, the unique sulfate transporter genes were upregulated, which was essential for sulfur absorption and distribution, maintaining cellular redox balance, and mitigating damage from reactive oxygen species. DEGs controlling classical hormonal signaling and adjusting the physiological responses under salinity, such as *GAs* (Gibberellins) and *ARF* (auxin response factor) family genes, were upregulated. *LEA* (late embryogenesis-abundant), a major ABA protein, influences the accumulation of osmolytes and stomatal closer to adjust water deficit conditions [74]. Understanding the functions of these endogenous hormones controlling numerous plant stages of development in acclimating chickpea adaptability to salinity requires the identification of genes that modulate these hormonal networks. Mainly, transcripts linked to transporters and signal transduction pathways were changed during stress conditions, indicating a putative role in salinity-adaptive phenotypes in tolerant genotypes (SSG 59-3), which is consistent with previous studies [75].

High salinity adversely affects the photosynthetic efficiency due to reductions in the saturation index, photosynthetic pigments, and quantum yield, and damage to the photosynthesis system. Photosynthetic enzymes, including ribulose bisphosphorous carboxylase (Rubisco), malate dehydrogenase, phosphoenolpyruvate carboxykinase (PEP-CK), and ferredoxin, are downregulated in salt-susceptible genotypes. In contrast, their upregulation in salt-tolerant genotypes might indicate the normal functioning of photosystem I and photosystem II to high salinity [76]. It is well documented that the unsaturation index in plants is reduced with the progression of salinity levels. Several genes participate in the *MAPK* processes, which are essential for the mechanism of oxidative stress. *SOS2*, a serine/threonine-protein kinase, was shown to interact with protein phosphatase 2C, and was suggested to play a key role in the salinity tolerance mechanism [77]. Under stressed environments, there is an elevated level of ethylene, leading to senescence. In this study, however, this SSG 59-3 salt tolerance capacity can be ascribed to the inhibition of ethylene biosynthesis.

The expression of genes encoding *NACs*, *WRKY*, *MYB*, and *AP2-EREBP* TFs were differentially regulated in sorghum genotypes (Supporting Figure S4). Previous reports have documented their involvement in response to high salinity [62,73] via the modulation of gene regulatory networks and cellular processes [78]. The TFs families engaged in hormone signaling, such as abscisic acid (ABA), auxins, cytokines, and gibberellins, were also expressed differentially, underlining the crucial role of plant growth regulators in response to salinity, as documented in other crops [79]. This study observed an upregulation of the DEGs that regulated *NACs*, *WRKY*, and *MYB* in the tolerant genotype (SSG 59-3). *NAC*s TFs impart salinity tolerance and were reported in several plant species, such as *Oryza sativa*, *Glycine max*, and *Cicer arietinum* [80,81]. *WRKY* transcription factors were induced to their maximum extent in tolerant genotypes. They were shown to be widely distributed in the plant genome, which regulates the downstream genes regulating responses to abiotic stresses, such as leaf senescence, phytohormones signaling and root biomass growth [82]. *MYB* TFs, an important class of transcription factors involved in primary/secondary metabolism and cell cycles, were substantially upregulated. By silencing the transcription, the *MYB* TFs can regulate both positive and negative gene expressions simultaneously. This is accompanied by expressing *MYB*-repressor genes involved in lignin biosynthesis and secondary cell wall formation [83]. *MYB* TFs, which play a key role in the primary or secondary metabolism and cell cycle, were also upregulated, while *AP2/ERF* TFs, as reported in this study, have a differential response to osmotic stress. Previous studies have shown that ethylene affects salt tolerance either positively or negatively. It regulates the expression of stress-related downstream genes and functions either as an activator or a repressor. In this study, the *ERF* gene was suppressed in the susceptible genotype and elevated in the tolerant genotype.

In response to high ROS, plants have developed antioxidative enzymatic (e.g., catalase, superoxide dismutase, and peroxidases) and non-enzymatic (e.g., glutathione cycles, ascorbate, and secondary metabolites) antioxidants in cells and tissues. Plants exposed to biotic and abiotic stresses generate excessive ROS in numerous cellular compartments, resulting in oxidative stress. Excess ROS activates Ca^2+^ and K^+^ permeable channels present in the plasma membrane, which further activates the Ca^2+^ cascade events and results in cell death [84]. The plant can detoxify excess ROS through its antioxidative machinery [8,9,10]. An ascorbate–glutathione cycle comprising several antioxidative enzymes, including L-ascorbate peroxidase, is among the most prominent strategies for eliminating hydrogen peroxide. Our work found that glutathione reductase, glutathione transferase, L-ascorbate peroxidase, catalase, and superoxide dismutase were also upregulated with elevated salt concentration is consistent with previous studies. Osmotic stress leads to ionic imbalance, damaging cellular membranes and disrupting imbalanced water homeostasis under high salinity [5,13]. To alleviate these toxic effects, the salt-tolerant genotype (SSG 59-3) accumulated several low molecular weight, compatible osmolytes, i.e., sugars (e.g., trehalose), proline, and betaine, which also helped to accommodate the excess ions in the vacuoles. The upregulation of the genes encoding for glycine betaine (GB), proline, and trehalose in sorghum reveals the accumulation of these compatible osmolytes to high salinity [84].

*NHX* proteins are ubiquitous transmembrane specialized proteins that maintain ion homeostasis by sequestering excess Na^+^ ions in vacuoles or eliminating from the cells. Proposing its role as a Na^+^/H^+^ repressor by downregulating *NHX* genes [85], crucial for the vascular compartmentalization of excess Na^+^ ions, it confers salt tolerance on diverse crop plants. In our study, the expression of genes encoding the Na^+^/H^+^ and H(^+^)-exporting diphosphatase exchangers, which are crucial for the transport of Na^+^/K^+^ and the maintenance of ion homeostasis, were upregulated in tolerant genotype. In a previous study, *AtABCG36*, the *Arabidopsis* ABC transporter, was also reported to enhance the adaptability of salinity stress by decreasing the sodium content in the shoots. Surprisingly, we identified an increased expression of different ATP-binding cassettes (ABC) and Na^+^/K^+^ transporter transcripts in sorghum samples at moderate/higher salinity.

Briefly, our study revealed comprehensive transcriptional reprogramming in addition to elucidating the roles of differentially expressed transcripts engaged in a variety of biological and cellular functions and metabolic processes, thereby explaining their function and regulatory mechanisms in chickpea salt tolerance, as discussed in previous transcriptome studies [85,86]. Additionally, this work gives valuable information and insight into the phenotypic and physio-biochemical behaviors of contrasting sorghum genotypes, making it possible to decipher the genetics underpinning salt tolerance. These findings may potentially pave the way for improving salt tolerance by exploiting suitable candidate genes and associated regulatory networks with the development of effective genome-assisted breeding strategies for the genetic improvement of sorghum.

## 5. Conclusions

This study provides a comprehensive transcriptome profiling of *Sorghum bicolor* and *SbNAC1* TFs under high salinity. A comprehensive comparison of RNAseq between the salt-tolerant (SSG 59-3) and susceptible (PC-5) genotype via the integration of physio-biochemical analysis facilitates better understanding the salt tolerance mechanism in sorghum. It offers a critical understanding of the molecular dynamics that underpin the metabolites, phytohormones, and gene regulatory networks. The GO and KEGG tools were used to evaluate the de novo transcriptome sequences, allowing annotation of a significant portion of the transcriptome. In response to increased salt concentrations, we observed that the DEGs encoding different TFs, ion transporters, antioxidants, osmolytes, photosynthesis genes, and aquaporins were upregulated, notably for signal transduction, osmotic homeostasis, and K^+^ ion transport and influx. Thresholds of sorghum-specific antioxidant defense mechanisms are stimulated at all developmental stages. Deciphering these specific alternatives could help developing more efficient metabolic engineering mechanisms, specific to different organs and ages, to cope with particular stress conditions. The results also indicated that the SSG 59- (salt-tolerant) genotype had used more efficient and comprehensive stress tolerance mechanisms via an active overexpression of candidate genes. Furthermore, real-time PCR (qPCR) analysis was used to validate the RNAseq data. This work will be instrumental in discerning molecular markers involved in salt stress response and may contribute to the improvement of knowledge about the plant kingdom. Additionally, this work may potentially pave the path to harness molecular breeding approaches in sorghum breeding programs in saline-prone areas.

## Figures and Tables

**Table 1:** Properties of soil in the pots under the screen house experiment.

Soil Texture	EC (%)	pH	OC (%)	N (mg/kg Soil)	P (mg/kg Soil)	K (mg/kg Soil)
Sandy-loam	0.09	7.50	0.18	86.33	3.67	165.0

**Table 2:** Description of primers used for real-time PCR.

Gene Symbol	Accession Number	Forward Primer (5′-3′)	Reverse Primer (5′-3′)
*SbGPX*	XM_002454470.2	CAAGGACCAGGGTTTTGAGA	GAGTGCAAGCAAACTGGACA
*SbLEA3*	GQ494000.1	CCGTTTGCTTGTTCAGGAGT	CAGCAACGGCGAATTAAACT
*SbP5CS1*	GQ377719.2	GTCACCAGATGAACGCAAAA	CCTCAACATCGGCTTCATTT
*SbBADH1*	U12195.1	AGCAGAAGCCTTGGACAAAA	AGCCCAACTACCCCCAATAG
*SbCIPK24*	XM_002438609.2	TCTCCAGGAGCCAAGTCATT	CAAACCATGGGTCTGCTCTT
*SbSNAC1*	KC253232.1	GACATGACCACCTCGCACT	GTTGTCCACGATCTCCGACT
*SbNCED3*	EER93751-1	CGAGAACTTCGTGGTCGTG	CGACGTCTTCTCCTTGTCCA
*SbH^+^-PPase*	GQ469975.1	GCTACGGCGACTACCTCATC	CCTTCGGAGATAGCGTTCTG
*SbPP2A*	XM_002448914.2	AAAAGGCTGCAGAAACGAAG	GCTTCAATTGGGGCAGATAA

**Table 3:** Read statistics of transcriptome sequencing in *Sorghum bicolor*.

Sample	Control	6 dSm^−1^	8 dSm^−1^	10 dSm^−1^	12 dSm^−1^
Raw reads	45,567,498	41,357,149	41,021,478	40,148,349	40,011,123
Processed clean reads	42,456,356	40,156,147	38,214,132	37,145,784	37,014,147
Alignment to clustered transcripts (%)	92.11	90.46	86.23	84.64	81.97
High quality reads (%)	90.37	85.95	83.95	83.56	80.74

## Data Availability

Data are contained within the article or Supplementary Material.

## Supplementary Materials

The following are available online at https://www.mdpi.com/article/10.3390/antiox10101605/s1, Table S1. Assembly statistics of unigenes in *Sorghum bicolor*; Figure S1. KEGG pathways of stress responsive metabolites. Pink represents highly upregulated metabolite biosynthetic pathways of the tolerant genotype and green represents those of the susceptible genotype; Figure S2. Phylogenetic relationships, gene structures, and conserved motifs of *SbSNAC1* TFs. (A) Phylogenetic tree of *SbSNAC1* proteins from soybean constructed using the ML method. (B) Exon/intron organization of *SbSNAC1* genes. Yellow boxes represent exons and black lines represent introns. The upstream/downstream regions of *SbSNAC1* genes are indicated by blue boxes. (C) Distribution of conserved motifs in *SbSNAC1* genes; Figure S3. The DNA binding domain (DBD) alignment of *SbSNAC1* TFs; Figure S4. (A) Comparisons of differentially expressed transcription factors in both the tolerant and the sensitive genotype under salt stress; (B) differentially expressed transcription factor families in sorghum genotypes under salt stress; Figure S5. Agarose gel: (A) mRNA of salt stress-responsive genes (1: *GPX*; 2: *LEA3*; 3: *CIPK24*; 4: *NCED3*; 5: *SNAC1*; 6: *H^+^-PPase*; 7: *Act*; 8: *BADH1*; 9: *P5CS1*; L: ladder); (B) PCR products of the expected sizes (≤ 200bp; 1: *Act*; 2: *LEA3*; 3: *CIPK24*; 4: *NCED3*; 5: *SNAC1*; 6: ^H+-^*PPase*; 7: *GPX*; 8: *PP2A*; 9: *BADH1*; 10: *P5CS1*; L: ladder); (C–J) melt/dissociation curve of salt stress-responsive genes under saline conditions; Figure S6. Expression stability and ranking of the salt responsive genes using: (A) geNorm, (B) NormFinder, and (C) comprehensive gene stability in *Sorghum bicolor* samples.
