# Supplementary material for: Genome-Wide Transcriptome Profiling, Characterization, and Functional Identification of NAC Transcription Factors in Sorghum under Salt Stress
Source: Antioxidants (Basel). 2021 Oct 13;10(10):1605. doi: 10.3390/antiox10101605 (PMC8533442; doi:10.3390/antiox10101605)
Supplement: Supplementary file 1 [file antioxidants-10-01605-s001.zip › antioxidants-1367202-supplementary.pdf]

Table S1. Assembly statistics of unigenes in *Sorghum bicolor.*

	Assembled transcripts	Clustered transcripts
**Number of transcripts identified**	171,896	125,457
**Maximum contig length**	13,459	13,459
**Minimum contig length**	350	350
**Average contig length**	1,145±1011.2	1,121
**Median contig length**	821.8	1436
**Total contigs length**	223,458,498	167,467,734
**Total number of non-ATGC characters**	0	0
**Contigs ≥ 200 bp**	174,698	121,563
**Contigs ≥ 500 bp**	132,459	97,462
**Contigs ≥ 1 Kbp**	79,164	48,627
**Contigs ≥ 10 Kbp**	28	19
**N50 value**	1845	1807

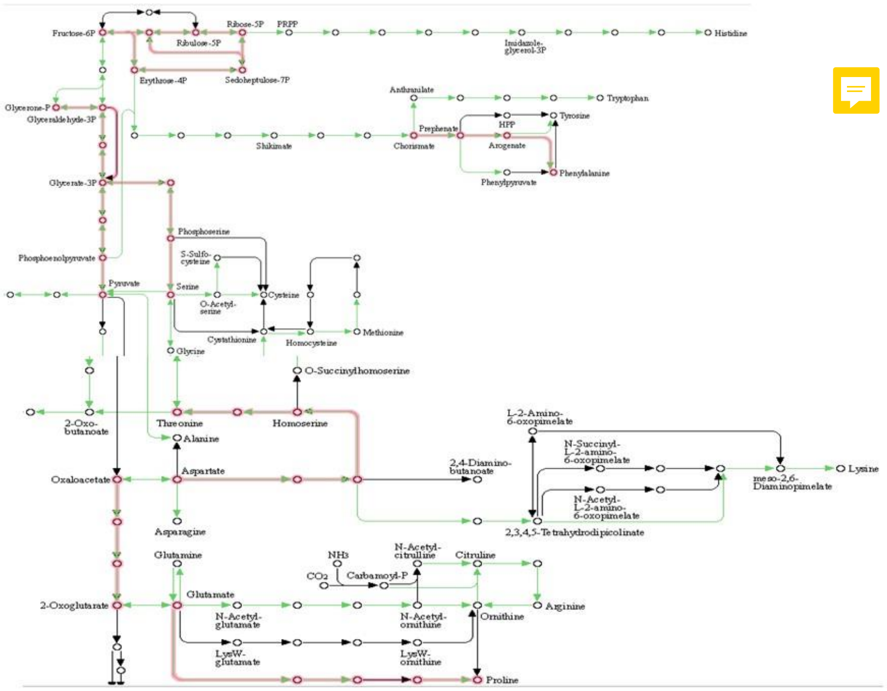

Figure S1. KEGG pathways of stress responsive metabolites. Pink represents highly upregulated metabolite biosynthetic pathways of the tolerant genotype and green represents those of the susceptible genotype.

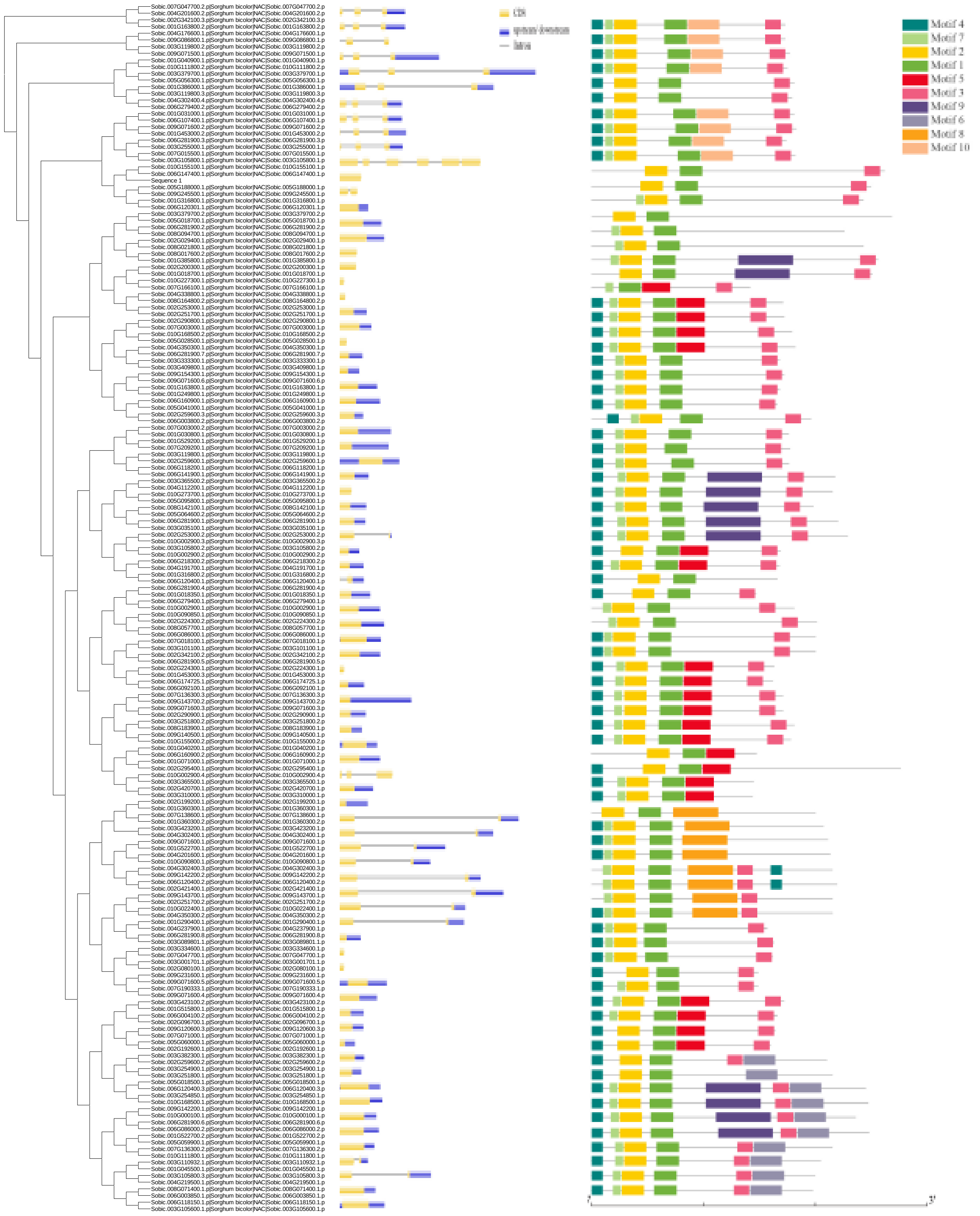

Figure S2. Phylogenetic relationships, gene structures, and conserved motifs of SbSNAC1 TFs. (A) Phylogenetic tree of *SbSNAC1* proteins from soybean constructed using the ML method.

(B) Exon/intron organization of *SbSNAC1* genes. Yellow boxes represent exons and black lines represent introns. The upstream/ downstream regions of *SbSNAC1* genes are indicated by blue boxes. (C) Distribution of conserved motifs in *SbSNAC1* genes.

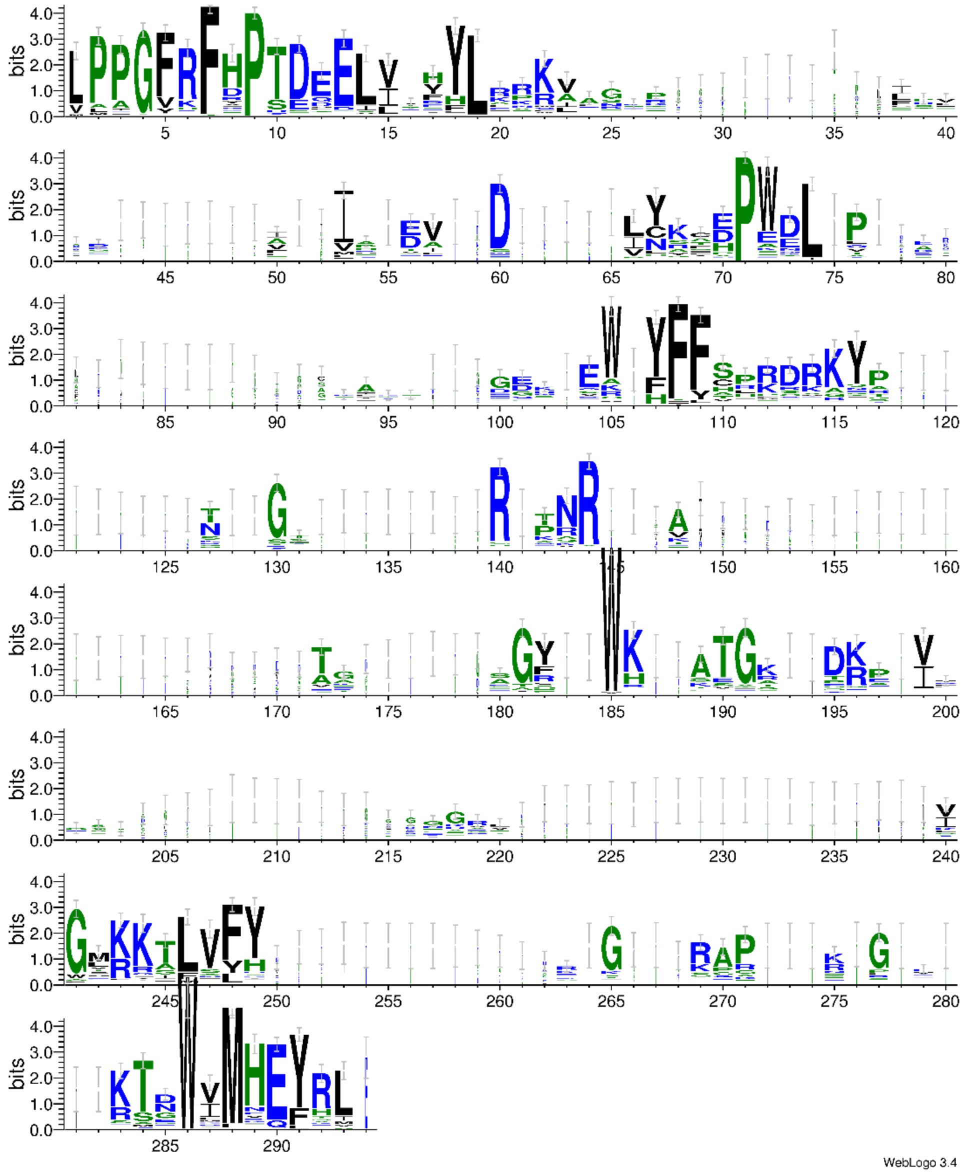

Figure S3. The DNA binding domain (DBD) alignment of *SbSNAC1* TFs.

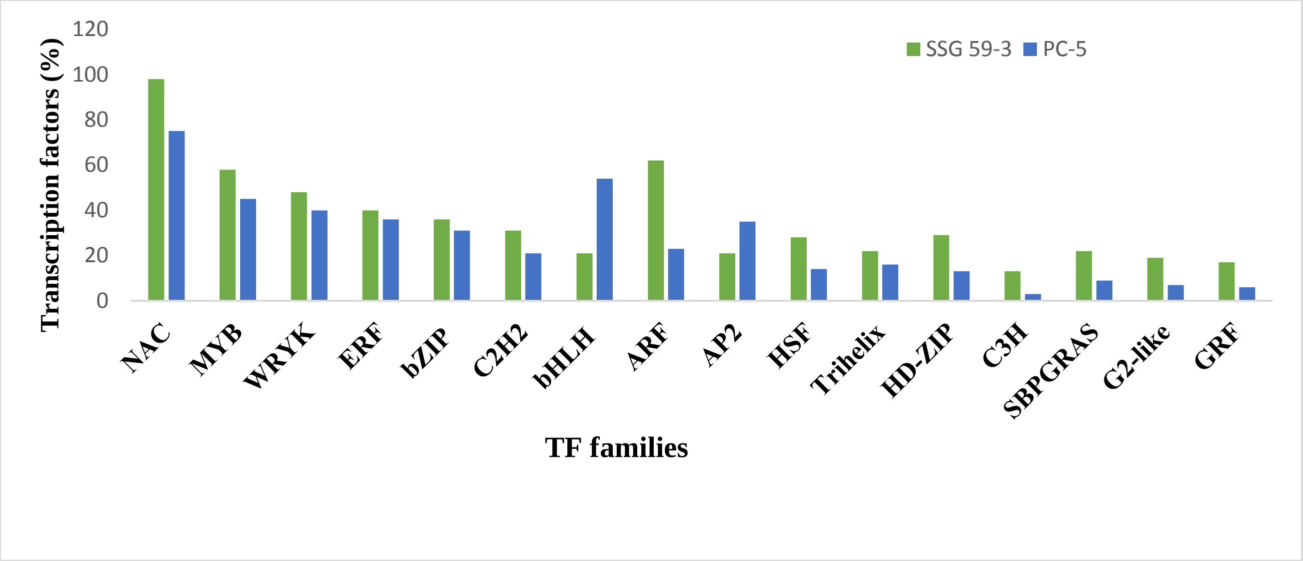

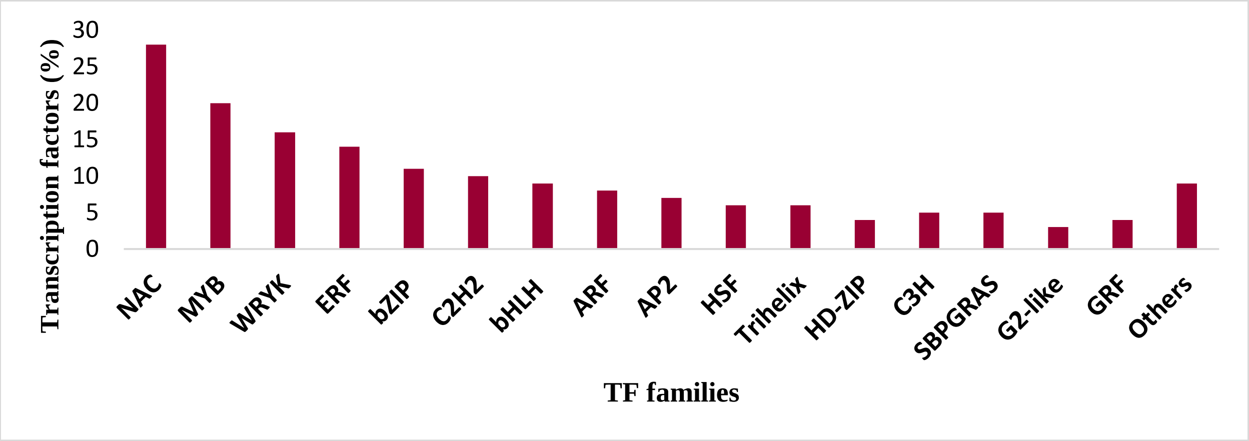

Figure S4. (A) Comparisons of differentially expressed transcription factors in both the tolerant and the sensitive genotype under salt stress; (B) differentially expressed transcription factor families in sorghum genotypes under salt stress.

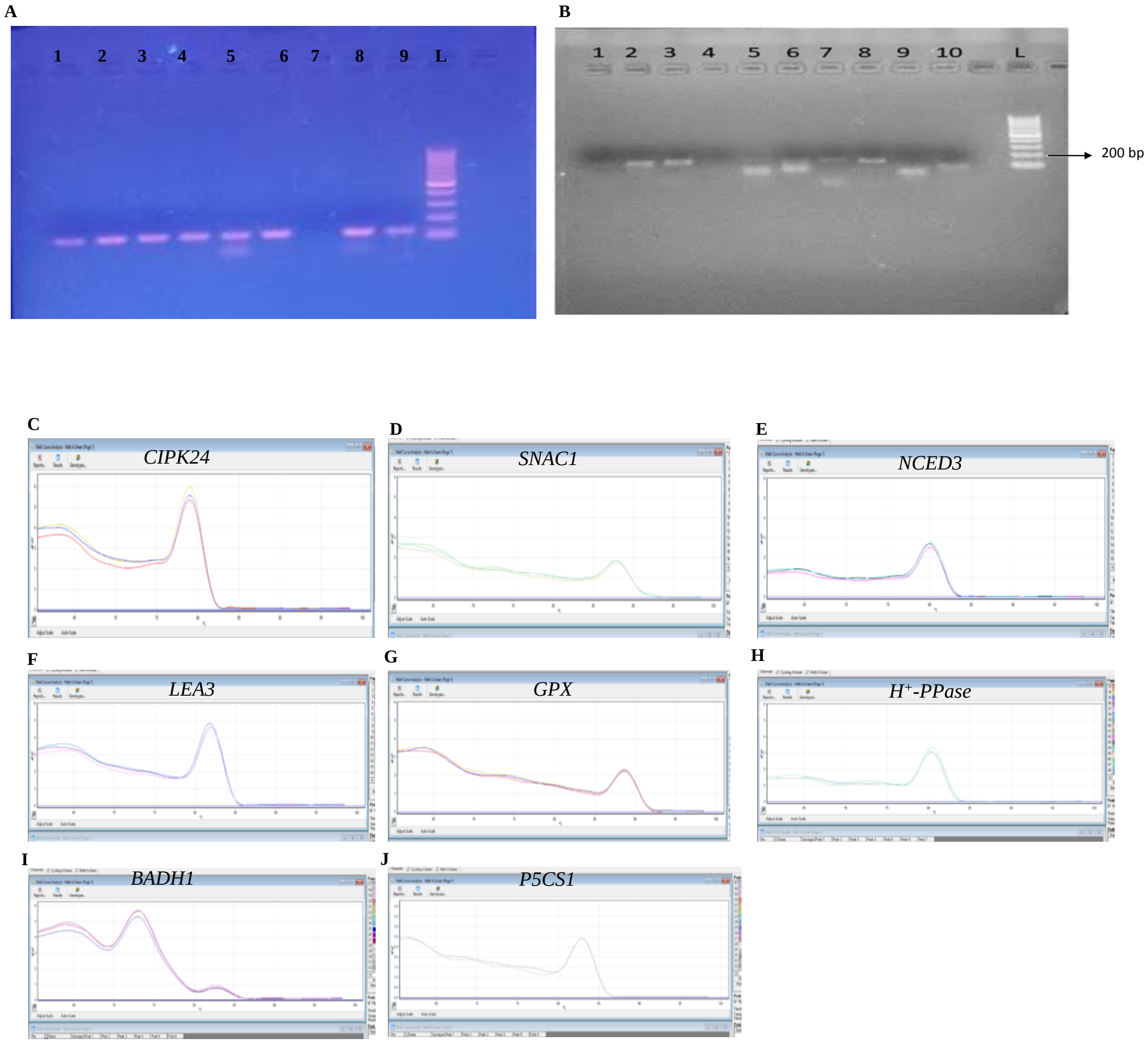

Figure S5. Agarose gel: (A) mRNA of salt stress-responsive genes ((1: *GPX*; 2: *LEA3*; 3: *CIPK24*; 4: *NCED3*; 5: *SNAC1*; 6: *H^+-PPase*; 7: *Act*;8: *BADH1*; 9: *P5CS1*; L: ladder), (B) PCR products of the expected sizes (≤ 200bp; 1: *Act*; 2: *LEA3*; 3: *CIPK24*; 4: *NCED3*; 5: *SNAC1*; 6: *H+-PPase*; 7: *GPX*; 8: *PP2A*; 9: *BADH1*; 10: *P5CS1*; L: ladder); (C–J) melt/dissociation curve of salt stress-responsive genes under saline conditions .

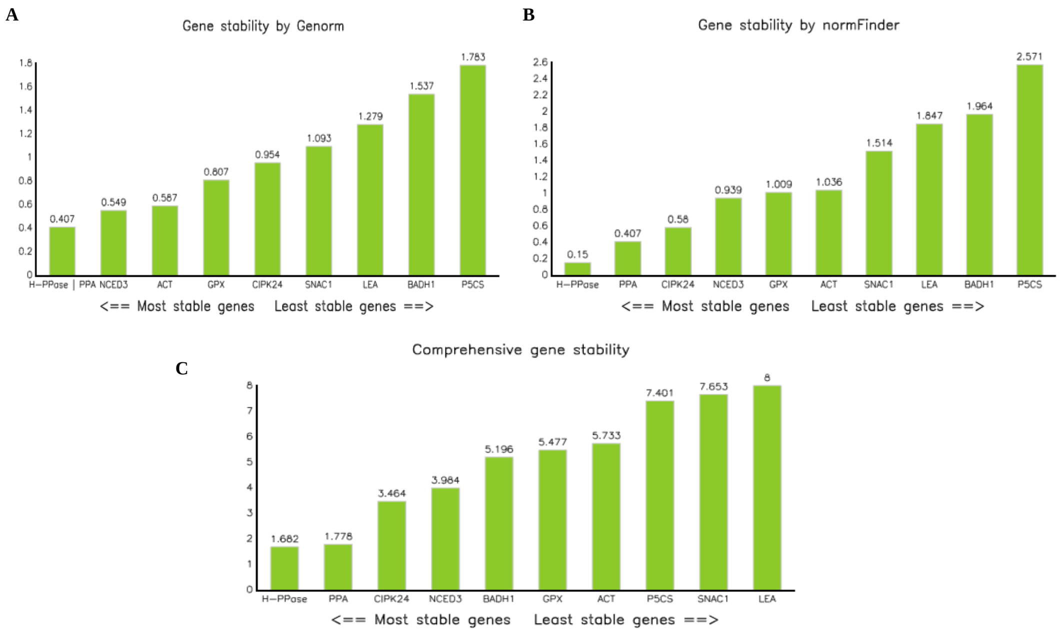

Figure S6. Expression stability and ranking of the salt responsive genes using: (A) geNorm, (B) NormFinder, and (C) comprehensive gene stability in *Sorghum bicolor* samples.
